# Evaluating and improving simulations of diurnal variation in land surface temperature with the Community Land Model for the Tibetan Plateau

**DOI:** 10.7717/peerj.11040

**Published:** 2021-03-16

**Authors:** Xiaogang Ma, Jiming Jin, Lingjing Zhu, Jian Liu

**Affiliations:** 1 Key Laboratory of Agricultural Soil and Water Engineering in Arid and Semiarid Areas, Ministry of Education, Northwest A&F Universtiy, Yangling, Shaanxi Province, China; 2 College of Water Resources and Architectural Engineering, Northwest A&F University, Yangling, Shaanxi Province, China; 3 South China Sea Institute of Marine Meteorology & College of Ocean and Meteorology, Guangdong Ocean University, Zhanjiang, Guangdong, China; 4 Southern Marine Science and Engineering Guangdong Laboratory, Zhanjiang, Guangdong, China

**Keywords:** Community land model version 5.0, Land surface temperature, Tibetan Plateau, Sensible heat roughness length, Soil thermal conductivity, Soil evaporation resistance

## Abstract

This study evaluated and improved the ability of the Community Land Model version 5.0 (CLM5.0) in simulating the diurnal land surface temperature (LST) cycle for the whole Tibetan Plateau (TP) by comparing it with Moderate Resolution Imaging Spectroradiometer satellite observations. During daytime, the model underestimated the LST on sparsely vegetated areas in summer, whereas cold biases occurred over the whole TP in winter. The lower simulated daytime LST resulted from weaker heat transfer resistances and greater soil thermal conductivity in the model, which generated a stronger heat flux transferred to the deep soil. During nighttime, CLM5.0 overestimated LST for the whole TP in both two seasons. These warm biases were mainly due to the greater soil thermal inertia, which is also related to greater soil thermal conductivity and wetter surface soil layer in the model. We employed the sensible heat roughness length scheme from Zeng, Wang & Wang (2012), the recommended soil thermal conductivity scheme from Dai et al. (2019), and the modified soil evaporation resistance parameterization, which was appropriate for the TP soil texture, to improve simulated daytime and nighttime LST, evapotranspiration, and surface (0–10 cm) soil moisture. In addition, the model produced lower daytime LST in winter because of overestimation of the snow cover fraction and an inaccurate atmospheric forcing dataset in the northwestern TP. In summary, this study reveals the reasons for biases when simulating LST variation, improves the simulations of turbulent fluxes and LST, and further shows that satellite-based observations can help enhance the land surface model parameterization and unobservable land surface processes on the TP.

## Introduction

The warming rate of the Tibetan Plateau (TP), known as Earth’s “Third Pole” (Qiu, 2008), has been significantly higher than the global average in the background of global warming in the past few decades (You et al., 2020). The unique and complex land surface processes of the TP strongly affect the interactions between the land surface and the atmosphere, which profoundly influence the formation and evolution of the Asian monsoon systems due to the mechanical and thermal forcing of the unique TP-topography (Duan et al., 2011; Fallah et al., 2016; Wang et al., 2019; Xue, Ma & Li, 2017). Thus, better understanding the land surface processes of the TP and more realistic descriptions of TP surface energy and water budgets are key parts of the study of TP land-atmosphere interactions (Fu et al., 2020; Gao et al., 2017; Lu et al., 2020; Wang et al., 2016). Land surface temperature (LST) determines the emission of surface longwave radiation, modulates sensible and latent heat fluxes according to the difference between LST and overlying atmospheric temperature (Wang et al., 2014), and affects the heat transfer between land surface and deep soil (Nogueira et al., 2020). These crucial processes describe the surface energy state, which is closely related to LST, which is an integrated variable that represents the energy and water exchanges between the land surface and the atmosphere (Deng et al., 2018; Jin & Dickinson, 2010; Johannsen et al., 2019; Nogueira et al., 2020). Hence, accurately understanding and characterizing LST of the TP are top priorities in improving the prediction of surface energy budget there (Babel, 2013; Gao et al., 2019; Jin & Mullens, 2012; Shi et al., 2019).

Previous studies about the LST of the TP used ground-based and satellite remote sensing observations. LST is not a regularly observed variable at meteorological stations (Wang et al., 2014), and these observations, with short data records, can be only acquired from limited stations of the TP, which are mainly located in the central and eastern parts. Thus, they cannot fully represent land surface states of the TP (Li et al., 2018; Oku et al., 2006; Qing-bai & Mao-cang, 2005; Zheng et al., 2015). LST derived from satellite remote sensing usually covers the entire TP, providing indispensable observed evidence over this data-sparse region. More specifically, LST products from the Moderate-Resolution Imaging Spectroradiometer (MODIS) are some of the best quality data (Phan & Kappas, 2018; Wan et al., 2017; Wan, 2008; Wang et al., 2007), with high temporal frequency (four daily satellite overpasses) and spatial resolution (500 m), and have been trustworthily employed as a surrogate for or a supplementary source to LST changes since 2000 (Jin & Mullens, 2012; Li et al., 2019a; Zhang et al., 2014; Zhong et al., 2010). However, it is difficult to fully understand the physical processes and mechanisms of LST changes and to quantitatively analyze the contributions of various elements to the TP land surface energy and water changes only by relying on remote sensing data (Chang et al., 2020; Ji, Yuan & Li, 2020). Therefore, additional tools are needed to conduct an in-depth investigation of LST variability on the TP.

Land surface models (LSMs) are a valuable tool used to produce long-term LST records in continuous spatiotemporal scales. LSMs are based on physical mechanism and parameterization schemes, providing the possibility to further understand the mechanisms related to LST changes (Hu et al., 2020; Orth et al., 2017; Trigo et al., 2015). LSMs usually generate LSTs that significant deviate from observations due to simplistic model representations of land surface heterogeneities, such as land surface cover type, soil properties, and soil moisture (Johannsen et al., 2019; Li et al., 2019a; Nogueira et al., 2020; Trigo et al., 2015; Wang et al., 2014). Many simulation results showed that the underestimation of LST and overestimation of sensible heat flux on bare-ground or sparsely vegetated surface during daytime are notable deficiencies in Noah LSM and Community Land Model version 3.5 (CLM3.5) (Zeng, Wang & Wang, 2012; Zheng et al., 2014). Misrepresentations of the green vegetation cover fraction in the Common Land Model (CoLM) and Carbon Hydrology Tiled ECMWF Scheme for Surface Exchanges over Land (CHTESSEL) produced warm and cold biases on dense vegetated surface during daytime, respectively (Li et al., 2019a; Nogueira et al., 2020).

Daytime errors of simulated LST and surface energy balance by LSMs have been effectively evaluated and modified. A new sensible heat roughness length scheme was developed on the basis of observations on bare ground (Chen et al., 2011; Yang et al., 2002), and simulations of LST and surface energy budget for arid area of China were improved in Noah LSM (Chen et al., 2011). Wang et al. (2014) compared LST from the CLM4.0 with in situ measurements and MODIS over the global scale and bare-ground surface, and reported that the modified ground surface sensible heat roughness length formulation (Zeng, Wang & Wang, 2012) reduced the model’s cold biases during daytime. Li et al. (2019a) adopted fractional vegetation cover schemes on the basis of the leaf area index (LAI) and a remotely sensed clumping index in CoLM, and significantly reduced the daytime warm LST bias over the TP’s grassland. Nogueira et al. (2020) improved the representation of vegetation over Iberia in CHTESSEL by combining the land cover with the LAI and a fraction of vegetation cover, and successfully completely removed the summer daytime cold LST bias. A few studies focused on nighttime LST processes with field observations, and found that simulated nighttime LST from CLM and Noah LSM was unrealistic under stable atmospheric stratification conditions (Zeng, Wang & Wang, 2012; Zheng et al., 2015). Obvious warm nighttime biases were dominant over most global land areas, especially in arid and semi-arid regions (Trigo et al., 2015; Wang et al., 2014). Unfortunately, these efforts had a negligible effect on the simulated nighttime LST errors (Wang et al., 2014; Zheng et al., 2015), although more striking warming trends were observed in nighttime than in the daytime on the TP (Duan & Wu, 2006; Jin & Mullens, 2012).

The objectives of this study were to (1) evaluate the diurnal LST cycle simulations in CLM version 5.0 (CLM5.0) on the TP using MODIS satellite products, (2) understand factors affecting LST errors in CLM5.0 simulations, and (3) systematically improve the ability of CLM5.0 simulations of diurnal LST cycle on the TP by introducing the ground sensible heat roughness length formulation from Zeng, Wang & Wang (2012), the recommended soil thermal conductivity scheme from Dai et al. (2019), which considers volumetric fractions of soil organic matter and soil gravel, and the modified soil evaporation resistance parameterization, which is appropriate for soil texture on the TP. In this paper, ‘Materials & Methods’ introduces the ground sensible heat roughness length, soil evaporation resistance, and soil thermal conductivity parameterization schemes in CLM5.0, and the LST calculation; describes the physical parameterization modifications to the CLM5.0; and provides information about the datasets and the design of the model’s numerical experiments. ‘Results’ presents the LST, evapotranspiration, and soil moisture simulation results, and compares them with observations that are further discussed in ‘Discussion’.

## Materials & Methods

### Model description

In this study, we used CLM5.0 (Lawrence et al., 2018) as the land surface model, which is the latest version developed by the National Center for Atmospheric Research and serves as the land surface model in the Community Earth System Model version 2.0. CLM5.0 is a “big-leaf” model that conceptualizes the vegetation canopy as a single layer, the snowpack is simulated with up to five layers depending on snow depth, and soil can be divided into an arbitrary number of layers (ten layers in this study). Compared with earlier versions of the model, CLM5.0 includes new soil evaporation resistance parameterization by introducing the concept of the dry surface layer (Swenson & Lawrence, 2014). In CLM5.0, each grid cell is split into different land units, including vegetated surfaces, lake, urban, glacier, and cropland. Each land-unit can be further split into several different columns. Each column is divided into multiple plant functional types (PFTs) (Bonan et al., 2002) or crop functional types. The spatial distribution and seasonal climatology of those PFTs for CLM5.0 are derived from MODIS satellite land surface data products (Lawrence & Chase, 2007). This sub-grid structure can help more accurately resolve the surface heterogeneity in complex terrain regions (Ma et al., 2019b).

In this study, we mainly focused on three parameterizations in CLM5.0: ground sensible heat roughness length (*z*_0*h*,*g*_), soil evaporation resistance (*r*_*soil*_), and soil thermal conductivity (*λ*), because our results indicated that these parameterizations strongly impact LST (discussed in detail in ‘Results’). Previous studies also reported similar findings (Chen et al., 2018; Pablos et al., 2016; Trigo et al., 2015; Wang et al., 2014; Zeng, Wang & Wang, 2012). Ground sensible heat roughness length (*z*_0*h*,*g*_) is of importance for the reliable calculation of the sensible heat flux from ground, which is a function of ground momentum roughness length (*z*_0*m*,*g*_):

(1) \begin{eqnarray*}{z}_{0h,g}={z}_{0m,g}\ast {e}^{-a({u}_{\ast }{z}_{0m,g}/\nu )^{b}}\end{eqnarray*}

where *z*_0*m*,*g*_ = 0.01 for soil and glacier, *z*_0*m*,*g*_ = 0.0024 for snow-covered surfaces (m); *u*_∗_ is the friction velocity, *ν* = 1.5∗10^−5^ m^2^ s^−1^ is the molecular viscosity, *a* = 0.13, and *b* = 0.45.

*r*_*soil*_ is used to represent the effect of soil resistance on soil evaporation, which is parameterized as:

(2a) \begin{eqnarray*}{r}_{soil}& = \frac{TDSL}{{D}_{\nu }\tau } \end{eqnarray*}

where *D*_*ν*_ is the molecular diffusivity of water vapor in air (m^2^ s^−2^), and *τ*describes the tortuosity of the vapor flow path through the soil matrix. These two parameters are related to soil type (Swenson & Lawrence, 2014). TDSL is the thickness of the dry surface layer (DSL, m) and given as:

(2b) \begin{eqnarray*}TDSL& = \left\{ \begin{array}{@{}l@{}} \displaystyle {T}_{\mathrm{max}}\ast \frac{{\theta }_{init}-{\theta }_{1}}{{\theta }_{init}-{\theta }_{air}} ({\theta }_{1}\lt {\theta }_{init})\\ \displaystyle 0 ({\theta }_{1}\geq {\theta }_{init}) \end{array} \right. \end{eqnarray*}

where *T*_max_= 15 is the maximal DSL thickness (mm); *θ*_*init*_ is the moisture value at which the DSL initiates and is equal to 0.8 times top model soil layer porosity, *θ*_1_ is the moisture value of the top model soil layer, and *θ*_*air*_ is the “air dry” soil moisture value (mm^3^ mm^−3^) (Lawrence et al., 2018).

In CLM5.0, the soil thermal conductivity (*λ*) is assumed to be a weighted combination of the saturated (*λ*_*sat*_) and dry (*λ*_*dry*_) thermal conductivity (Lawrence et al., 2018):

(3a) \begin{eqnarray*}\lambda & = \left\{ \begin{array}{@{}l@{}} \displaystyle {K}_{e}\ast {\lambda }_{sat}+(1-{K}_{e})\ast {\lambda }_{dry} ({S}_{r}\gt 1{0}^{-7})\\ \displaystyle {\lambda }_{dry} ({S}_{r}\leq 1{0}^{-7}) \end{array} \right. \end{eqnarray*}

where *K*_*e*_ is the Kersten number expressed as a function of water phase and saturation degree (*S*_*r*_ = *θ*∕*ϕ*, *θ* is the real soil moisture, *ϕ* is the soil porosity):

(3b) \begin{eqnarray*}{K}_{e}& = \left\{ \begin{array}{@{}l@{}} \displaystyle l{\mathrm{og}}_{10}({\mathrm{S}}_{\mathrm{r}})+1 ({T}_{soil}\geq {T}_{f})\\ \displaystyle {\mathrm{S}}_{\mathrm{r}} ({T}_{soil}\lt {T}_{f}) \end{array} \right. \end{eqnarray*}

Dry soil thermal conductivity (*λ*_*dry*_) is estimated using the weighted mean of the thermal conductivities of dry mineral soil and dry soil organic matter (SOM), respectively:

(3c) \begin{eqnarray*}{\lambda }_{dry}& =(1-{f}_{om})\ast {\lambda }_{dry,m}+{f}_{om}\ast {\lambda }_{dry,om}\end{eqnarray*}

where *f*_*om*_ = *ρ*_*om*_∕*ρ*_*om*,max_ is the SOM fraction, *ρ*_*om*_ is the SOM density (kg m^−3^) acquired from input surface data, *ρ*_*om*,max_= 130 kg m^−3^ is the bulk density of peat. *λ*_*dry*,*om*_ = 0.05 W m^−1^ K^−1^ is the dry SOM thermal conductivity, and *λ*_*dry*,*m*_ is the dry mineral soil thermal conductivity (W m^−1^ K^−1^), which depends on bulk density *ρ*_*d*_= 2700 ∗(1 − *ϕ*)(kg m^−3^) as:

(3d) \begin{eqnarray*}{\lambda }_{dry,m}& = \frac{0.135\ast {\rho }_{d}+64.7}{2700-0.947\ast {\rho }_{d}} \end{eqnarray*}

Saturated thermal conductivity (*λ*_*sat*_) depends on the thermal conductivities of the soil solid, liquid water, and ice constituents:

(3e) \begin{eqnarray*}{\lambda }_{sat}& ={\lambda }_{s}^{1-\phi }\ast {\lambda }_{liq}^{ \frac{{\theta }_{liq}}{{\theta }_{liq}+{\theta }_{ice}} \ast \phi }\ast {\lambda }_{ice}^{(1- \frac{{\theta }_{liq}}{{\theta }_{liq}+{\theta }_{ice}} )\ast \phi }\end{eqnarray*}

where *θ*_*liq*_ and *θ*_*ice*_ are the soil liquid water and ice contents (mm^3^ mm^−3^), respectively; *λ*_*liq*_ = 0.57 W m^−1^ K^−1^ and *λ*_*ice*_ = 2.29 W m^−1^ K^−1^ are liquid water and ice thermal conductivities, respectively; and *λ*_*s*_ is the soil solid thermal conductivity, which is calculated using the weighted mean of thermal conductivities of mineral soil and SOM:

(3f) \begin{eqnarray*}{\lambda }_{s}& =(1-{f}_{om})\ast {\lambda }_{s,m}+{f}_{om}\ast {\lambda }_{s,om}\end{eqnarray*}

where *λ*_*s*,*om*_ = 0.25 W m^−1^ K^−1^ is the SOM thermal conductivity and *λ*_*s*,*m*_ isthe mineral soil solid thermal conductivity:

(3g) \begin{eqnarray*}{\lambda }_{s,m}& = \frac{8.80\ast (\text{%}sand)+2.92\ast (\text{%}clay)}{(\text{%}sand)+(\text{%}clay)} \end{eqnarray*}

where %sand and %clay represent the gravimetric fractions of sand and clay in mineral soil, respectively, and are acquired from the input surface data.

### LST calculation

LST is calculated with upward land surface longwave radiation (*L*↑), downward longwave radiation (*L*↓), and land surface emissivity (ε) as follows (Wang et al., 2014):

(4) \begin{eqnarray*}LST=\sqrt[4]{ \frac{L\uparrow -(1-\varepsilon )\ast L\downarrow }{\varepsilon \ast \sigma } }\end{eqnarray*}

where *σ*= 5.67*10^−8^ W m^−2^ K^−4^ is the Stefan–Boltzmann constant. Atmospheric downward longwave radiation *L*↓ was obtained from the atmospheric forcing dataset (see ‘Climate forcing data’). Surface upward longwave radiation *L*↑ is the areally-weighted value with the fractions of all the PFTs in the grid cell of CLM5.0. Land surface emissivity (ε) was calculated with the ground emissivity (ε_*g*_) and vegetation emissivity (ε_*v*_) from CLM5.0:

(5) \begin{eqnarray*}\varepsilon ={\varepsilon }_{v}+{\varepsilon }_{g}\ast (1-{\varepsilon }_{v})+{\varepsilon }_{v}\ast (1-{\varepsilon }_{g})\ast (1-{\varepsilon }_{v})\end{eqnarray*}

where ground emissivity ε_*g*_ is combined with the snow emissivity and the soil emissivity weighted by the snow cover fraction. Vegetation emissivity ε_*v*_ is parameterized with the exposed leaf and stem area indices (Lawrence et al., 2018). In this study, we used satellite data to evaluate the calculated LST with Eq. (4).

### Modifications for three parameterizations

#### Revision of ground sensible heat roughness length scheme

*z*_0*h*,*g*_ is an important parameter used to estimate ground sensible heat flux in many existing LSMs (Chen et al., 2011; Trigo et al., 2015; Zheng et al., 2015). Zeng, Wang & Wang (2012) revised *z*_0*h*,*g*_ parameterization in CLM3.5 for two semiarid sites, and reported significantly improved daytime LST simulations over bare ground areas. This revision of *z*_0*h*,*g*_can be expressed as:

(6) \begin{eqnarray*}\ln \nolimits ( \frac{{z}_{0m,g}}{{z}_{0h,g}} )=a\ast ( \frac{{u}_{\ast }{z}_{0m,g}}{\nu } )^{b}\end{eqnarray*}

where *a* = 0.36 and *b* = 0.5.

#### Modification scheme for soil thermal conductivity

Both Dai et al. (2019) and we (‘Impact of soil thermal conductivity on LST’) recognized that the original *λ* parameterization in CLM5.0 produces a larger *λ* when compared with in situ observations. Thus, on the basis of these conclusions and the recommendation of Dai et al. (2019), modified soil thermal conductivity parameterization was adopted in this study, which mainly originated from Johansen (1977):

(7a) \begin{eqnarray*}\lambda & = \left\{ \begin{array}{@{}l@{}} \displaystyle {K}_{e}\ast {\lambda }_{sat}+(1-{K}_{e})\ast {\lambda }_{dry} ({S}_{r}\gt 1{0}^{-7})\\ \displaystyle {\lambda }_{dry} ({S}_{r}\leq 1{0}^{-7}) \end{array} \right. \end{eqnarray*}

where *K*_*e*_ is also a function of the phase of water and the saturation degree *S*_*r*_, but the exponential form of *S*_*r*_ was applied to express *K*_*e*_ to avoid the negative values (Yang et al., 2005):

(7b) \begin{eqnarray*}{K}_{e}& = \left\{ \begin{array}{@{}l@{}} \displaystyle e\mathrm{xp}[0.36\mathrm{ \ast }(1-1/{\mathrm{S}}_{\mathrm{r}})]\, ({T}_{soil}\geq {T}_{f})\\ \displaystyle {\mathrm{S}}_{\mathrm{r}} ({T}_{soil}\lt {T}_{f}) \end{array} \right. \end{eqnarray*}

the dry soil thermal conductivity *λ*_*dry*_ is calculated as:

(7c) \begin{eqnarray*}{\lambda }_{dry}& ={V}_{m}\ast {\lambda }_{dry,m}+{V}_{om}\ast {\lambda }_{dry,om}+{V}_{g}\ast {\lambda }_{dry,g}\end{eqnarray*}

where *λ*_*dry*,*m*_ is calculated using the Eq. (3d); *λ*_*dry*,*g*_ is the soil gravel thermal conductivity in dry conditions, and estimated empirically as *λ*_*dry*,*g*_= 0.039 ∗*ϕ*^−2.2^; *V*_*m*_, *V*_*om*_, and *V*_*g*_ are the volumetric fractions of mineral soils, SOM, and gravels in soil solids, respectively. Here, *V*_*om*_ and *V*_*g*_ were calculated on the basis of SOM mass content and gravel mass proportion according to Chen et al. (2012):

(7d) \begin{eqnarray*}{V}_{om}& = \frac{{\rho }_{p}(1-{\phi }_{m}){m}_{om}}{{\rho }_{om,\max \nolimits }(1-{m}_{om})+{\rho }_{p}(1-{\phi }_{m}){m}_{om}+(1-{\phi }_{m})\ast \frac{{\rho }_{om,\max \nolimits }{m}_{g}}{(1-{m}_{g})} } \end{eqnarray*}

(7e) \begin{eqnarray*}{V}_{g}& = \frac{{\rho }_{om,\max \nolimits }(1-{\phi }_{m}){m}_{g}}{(1-{m}_{g})[{\rho }_{om,\max \nolimits }(1-{m}_{om})+{\rho }_{p}(1-{\phi }_{m}){m}_{om}+(1-{\phi }_{m})\ast \frac{{\rho }_{om,\max \nolimits }{m}_{g}}{(1-{m}_{g})} ]} \end{eqnarray*}

where *ρ*_*p*_= 2700 kg m^−3^ is the mineral particle density. *ϕ*_*m*_= 0.489–0.0012 ∗(%*sand*) is the mineral soil porosity (mm^3^ mm^−3^). *m*_*om*_ and *m*_*g*_are the SOM and soil gravel mass proportions, respectively, acquired from input surface data. Saturated soil thermal conductivity *λ*_*sat*_ is calculated as:

(7f) \begin{eqnarray*}{\lambda }_{sat}& ={\lambda }_{s,m}^{{V}_{m}}\ast {\lambda }_{s,om}^{{V}_{om}}\ast {\lambda }_{g}^{{V}_{g}}\ast {\lambda }_{w}^{\phi }\end{eqnarray*}

where *λ*_*s*,*m*_ and *λ*_*g*_ are the mineral soil solid and soil gravel thermal conductivities in wet conditions, respectively. Here, *λ*_*s*,*m*_ depends on quartz content *V*_*q*_, which is commonly considered to be equal to 50% of the sand content in this study (Chen et al., 2012; Luo et al., 2017) and can be estimated with the following:

(7g) \begin{eqnarray*}{\lambda }_{s,m}& ={\lambda }_{q}^{{V}_{q}}\ast {\lambda }_{o}^{1-{V}_{q}}\end{eqnarray*}

where *λ*_*q*_ = 7.7 W m^−1^ K^−1^ is the quartz thermal conductivity; other non-quartz minerals thermal conductivity is given as *λ*_*o*_ = 2.0 W m^−1^ K^−1^ for *V*_*q*_ >0.2 and *λ*_*o*_ = 3.0 W m^−1^ K^−1^ otherwise. *λ*_*g*_ as assigned the same value as that of the dry condition (0.039 ∗*ϕ*^−2.2^). *λ*_*w*_ = 0.57 W m^−1^ K^−1^ for unfrozen water status and *λ*_*w*_ = 2.29 W m^−1^ K^−1^ for frozen water status.

#### Modification of soil evaporation resistance scheme

An underestimation of soil evaporation and a prediction of wetter surface soil are produced by the original CLM5.0 in summer (‘Impact of soil evaporation resistance on LST’). These biases are caused by the overestimation of soil evaporation resistance (*r*_*soil*_ in Eq. (2a)). Soil evaporation resistance is positively related to the TDSL, which is parameterized as a function of top soil layer moisture and soil type (Eq. (2b)). Here, the moisture value, *θ*_*init*_, plays an important role in determining TDSL (or soil evaporation resistance) for a given soil type. This original moisture value (*θ*_*init*_) is taken as 0.8 times soil porosity, which is suitable for typical loam soil (34% sand, 24% clay; (Swenson & Lawrence, 2014). However, Lehmann et al. (2018) stated that the coarser the soil texture, the lower *θ*_*init*_value for the soil evaporation. Top surface soil contains more sand (above 60%) and less clay (below 20%) in the central and western TP (Fig. S1), which implies that the original value of *θ*_*init*_ (0.8 times soil porosity) is not appropriate to these sandier soils. Van de Griend & Owe (1994) conducted a field experiment with a fine sandy loam soil (69% sand, 11% clay), and found that zero soil surface resistance occurred at a *θ*_*init*_ of approximately 0.15 mm^3^ mm^−3^ (about 0.37 times soil porosity). Other field observations and theoretical analysis also demonstrated that sandier soils have a lower *θ*_*init*_value than that of clay soil (Bittelli et al., 2008; Lehmann et al., 2018; Yamanaka & Yonetani, 1999). In addition, maximal DSL thickness was set to 20 mm on the basis of field observations, which is suitable for both sandy and clay soils (Yamanaka, Takeda & Shimada, 2006). Therefore, *T*_max_= 20 mm and *θ*_*init*_ = 0.37 times soil porosity were applied into CLM5.0.

### Data

#### Climate forcing data

In our study, the China Meteorological Forcing Dataset (CMFD) (He et al., 2020) was used to drive the CLM5.0. This dataset includes seven atmospheric variables: air temperature (K), air pressure (Pa), specific humidity (kg kg^−1^), wind speed (m s^−1^), downward shortwave and longwave radiation (W m^−2^), and precipitation rate (mm s^−1^). The CMFD was produced by merging ground-based observations with several gridded datasets from remote sensing and reanalysis data. Ground-based observations were obtained from 753 meteorological stations owned by the China Meteorological Administration (CMA). The gridded remote sensing/reanalysis data included several data sources: Global Land Data Assimilation System and Modern Era Retrospective-Analysis for Research and Applications data, Tropical Rainfall Measuring Mission satellite precipitation analysis data, and Global Energy and Water Cycle Experiment-Surface Radiation Budget downward shortwave radiation data, downward longwave radiation was calculated by using a model described in Crawford & Duchon (1999). The CMFD record begins in January 1979 and is currently at December 2018, with a 3-hour time step, 0.1° spatial resolution, and coverage of the entire area of China. The dataset has been verified by many studies and used for hydrological modeling, regional climate simulations validation, and land data assimilation (Li et al., 2019a; Li et al., 2018; Tian et al., 2020; Zhu, Jin & Liu, 2020).

#### Soil gravel data

A gridded soil gravel mass proportion dataset (Shangguan et al., 2013) was introduced into CLM5.0 to more reasonably characterize the soil properties of the TP. The gridded soil gravel mass proportion dataset was embedded in the soil characteristics dataset of China, which was derived from 8,979 soil profiles and the soil map of China through the polygon linkage method under the China Genetic Soil Classification framework, and covered the entire main land of China with 30 × 30 arc-second spatial resolution. The dataset provides complete, high precision soil properties that can be used as input parameters for LSMs, and has been widely used for regional land surface modeling (Bi et al., 2016) and biogeochemical research (Lu et al., 2018a; Zhang et al., 2019).

#### Validation data

In this study, we assessed the simulated diurnal LST cycle and emissivity against the MODIS/Aqua LST and emissivity daily level 3 global 0.05° Climate Modeling Grid products (MYD11C1, Version 6) (Wan, 2013). The daytime overpass time is around 1:30 p.m. (ascending mode, local solar time), which is closer to the time when relatively high LST values occur in the diurnal cycle. Thus, the daytime MODIS/Aqua MYD11C1 LST data were adopted as the primary validation data (Chen et al., 2011; Li et al., 2019a). In addition, we choose the MODIS/Aqua nighttime (around 1:30 a.m., local solar time) LST data to examine the performance of the simulated diurnal LST cycle. Many previous studies validated the accuracy of MODIS LST and emissivity products through long-term ground-based observations, which proved to be highly accurate (Jin & Liang, 2006; Lu et al., 2018b; Wang et al., 2007). Then, they were applied to observational and modeling studies on regional and global scales, including the TP region (Chen et al., 2011; Jin & Mullens, 2012; Li et al., 2019a; Ma et al., 2019b; Wang et al., 2014). In this study, only the highest-quality MODIS LST data (LST mean errors less than 1 K) marked in Wan (2013) were selected in both daytime and nighttime. Only the highest-quality retrieved individual spectral bands highest-quality MODIS emissivity values (emissivity mean errors less than 0.01) for wavelengths of 8.40–8.70 µm (Band 29), 10.78–11.28 µm (Band 31), and 11.77–12.27 µm (Band 32) were integrated to calculate the broadband emissivity (8–14  µm) on the basis of the method described in Wang & Liang (2009), which are consistent with the broadband emissivity needed by LSMs (Ma et al., 2019b; Sobrino, Jiménez-Muñoz & Verhoef, 2005). In addition, we interpolated the MODIS LST and calculated broadband emissivity data at a 0.05° spatial resolution onto 0.1° grids coincident with the spatial resolution of the model output.

In this paper, the Global Land Evaporation Amsterdam Model (GLEAM) version 3.2b dataset was chosen as the validation data for simulated evapotranspiration (ET) and soil moisture (SM). The GLEAM dataset provides global gridded estimates of different ET components, and surface (0–10 cm) soil moisture based on satellite observations with a spatial resolution of 0.25° longitude and 0.25° latitude, and a daily temporal resolution (Martens et al., 2017). GLEAM uses the Priestley and Taylor equation to calculate potential evaporation on the basis of the satellite observations of net surface radiation and near-surface air temperature. Estimations of potential evaporation are converted into actual evaporation on the basis of the evaporative stress factor. In addition, observations of surface soil moisture were assimilated into the dataset. This dataset was successfully validated with ground measurements of evaporation and soil moisture across global stations, showing good performance in all vegetation types and climate conditions (Gonzalez Miralles et al., 2011; Li et al., 2019b; Miralles et al., 2014). It was then extensively applied for evaluation of terrestrial evaporation and soil moisture responses to climate change (Guillod et al., 2015; Martens et al., 2018; Schumacher et al., 2019).

We collected in situ observations from the Ngari Station for Desert Environment Observation and Research (NASDE, 33.39°N, 79.7°E) (Ma et al., 2008) to evaluate the quality of the CMFD forcing data. Field observations from the BJ site (31.37°N, 91.90°E) (Liu et al., 2020) on the TP were used to assess the simulated soil thermal conductivity. Basic BJ site information, including soil texture, SOM, and soil gravel mass proportion is listed in Table 1 (Chen et al., 2012; Pan et al., 2017). BJ provides half-hourly atmospheric forcing data, soil temperature and soil moisture profiles observed at depths of 4 and 20 cm, and soil heat flux measured at depths of 10 and 20 cm. Here, the half-hourly 10 cm soil thermal conductivity measurements were calculated on the basis of the Eq. (8), and we rejected observations with an absolute value was less than 5 W m^−1^ K^−1^, which indicated an unstable soil thermal condition (Luo et al., 2009):

**Table 1:** Soil texture, soil organic, and soil gravel content at BJ station (31.37°N, 91.90°E).

Soil depth (m)	Sand (%)	Clay (%)	*m*_*om*_ (%)	*m*_*g*_(%)
0.0175	63.68	4.13	2.1	10
0.0451	63.68	4.13	2.1	10
0.0906	63.68	4.13	2.1	10
0.1656	63.68	4.13	1.3	10
0.2891	43.50	10.99	1.1	10
0.4930	71.94	3.58	1.5	19.03
0.8289	67.08	0.88	1.5	28.46
1.3828	64.75	1.87	1.5	28.46
2.2961	64.75	1.87	0	28.46
3.4331	64.75	1.87	0	28.46

(8) \begin{eqnarray*}{\lambda }_{10cm}= \frac{{G}_{10cm}\ast \Delta z}{{T}_{4cm}-{T}_{20cm}} \end{eqnarray*}

where *G*_10*cm*_ is the soil heat flux measured at a depth of 10 cm, Δz=0 .16 m, and *T*_4*cm*_, *T*_20*cm*_ are the soil temperature measured at depths of 4 and 20 cm, respectively.

### Design of numerical experiments

A suite of numerical experiments was performed to assess the response of the simulated LST, soil thermal properties, and turbulent fluxes to the different representations of ground sensible heat roughness length, soil thermal conductivity, and soil evaporation resistance parameterizations with CLM5.0. Four offline CLM5.0 simulations for the TP for 1995–2018 were conducted with the prescribed satellite-derived phenology. Here, these simulations were conducted only for the TP in China due to the atmospheric forcing dataset CMFD only covering China. CLM5.0 was first run with the original parameterizations (‘Model Description’), defined as the control run (CTL). The second simulation, which employed revision *z*_0*h*,*g*_ (described in ‘Revision of ground sensible heat roughness length scheme’), was denoted as EXP1. The third experiment (EXP2) duplicated EXP1, but replaced the original soil thermal conductivity scheme with the modified scheme that was described in ‘Modification scheme for soil thermal conductivity’, On the basis of EXP2, soil evaporation resistance parameterization was improved as described in ‘Modification of soil evaporation resistance scheme’ (EXP3). The different experimental setups are summarized in Table 2. The spatial resolution of these four simulations was 0.1°, the same as that of the atmospheric forcing dataset, which reduced the error due to horizontal interpolation. Hourly model outputs for the period of December 2002 through November 2018 were used in the following analysis. The calculated hourly LST was interpolated to the two MODIS Aqua satellite overpass times (local solar time 1:30 a.m. and 1:30 p.m.) and then compared with MODIS Aqua LST. In this study, December, January, and February were defined as winter; and June, July, and August were denoted as summer. In addition, we employed statistical metrics such as the spatial pattern correlation coefficient (PCC), root-mean-square-error (RMSE), and average bias to evaluate the performance of CLM5.0.

**Table 2:** List of numerical experiments designed to test modifications for the CLM5.0.

Experiment	*z*_0*h*,*g*_	*λ*	*r*_*soil*_
CTL	Original	Original	Original
EXP1	Equation (6)	Original	Original
EXP2	Equation (6)	Equation (7a-7g)	Original
EXP3	Equation (6)	Equation (7a-7g)	*T*_max_= 20 mm *θ*_*init*_= 0.37* *ϕ*

## Results

### Evaluation of the CTL simulation

**Figure 1:**
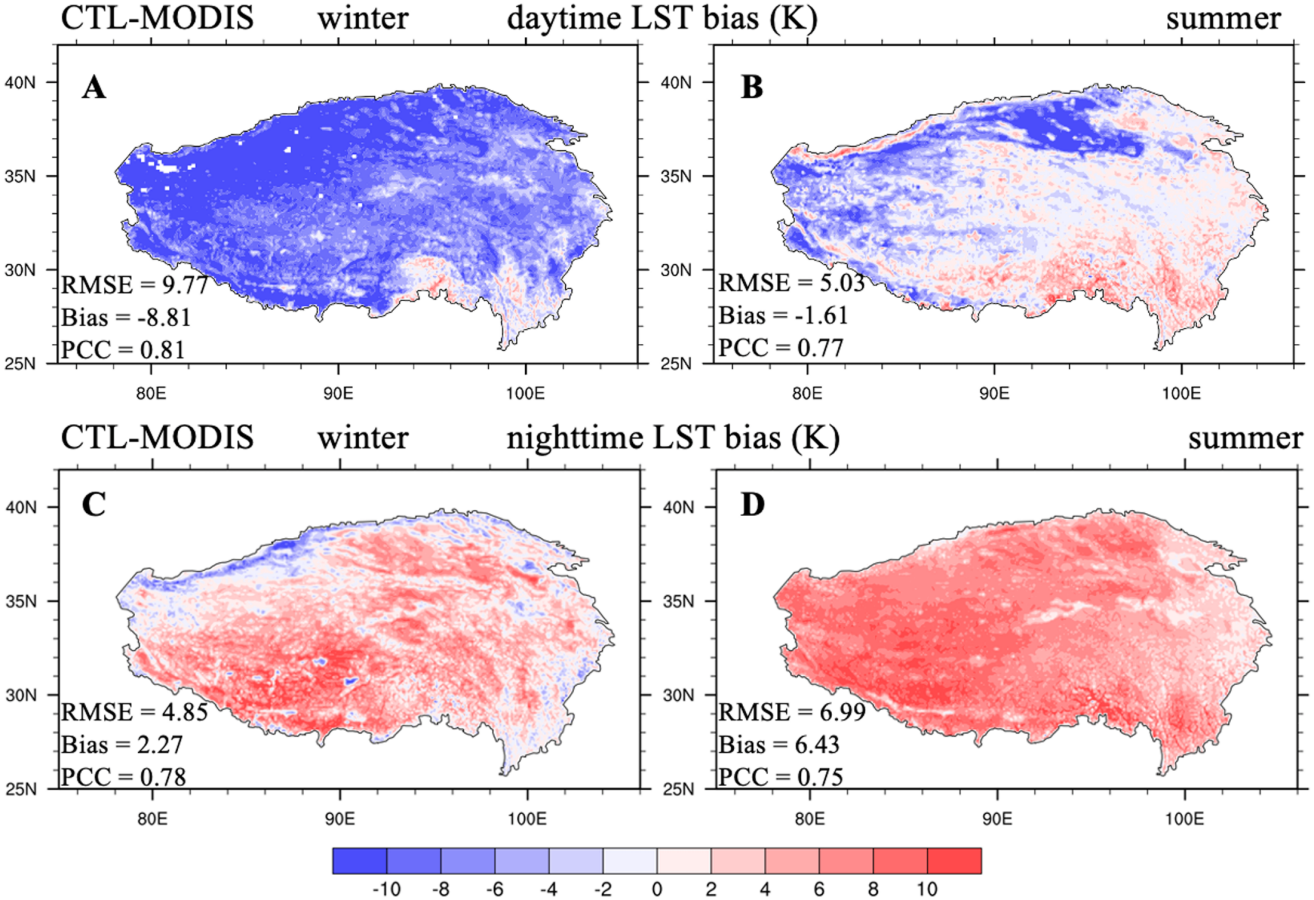
Seasonal distributions of (A, B) daytime and (C, D) nighttime LST biases (unit: K) between CTL and MODIS/Aqua (CTL-MODIS) averaged over 2003–2018 for winter and summer.

We first present the performance of CLM5.0 with the original parameterizations (CTL) compared with MODIS LST products. Figure 1 shows the spatial distribution of the LST biases between the CTL and MODIS Aqua data averaged over 2003-2018 during daytime and nighttime in summer and winter, respectively. The LST biases displayed large spatial and diurnal variations. During daytime, the model produced cold biases for almost the entire TP region in winter (Fig. 1A), with a −8.81 K average bias and an RMSE of 9.77 K. In summer, cold biases appeared mainly on bare-ground regions (western TP and the Qaidam Basin), whereas warm biases mainly appeared on vegetated regions (southern TP; Fig. 1B). In addition, the average temperature bias was −1.61 K and RMSE was 5.03 K, averaged for the TP in summer. During nighttime, a warm bias covered the entire TP for both two seasons, whereas the cold bias was less pronounced over the northwestern TP in winter (Figs. 1C, 1D). The average nighttime LST for the TP varied from 2.27 K in winter to 6.43 K in summer. CLM5.0 reasonably captured the spatial patterns of LST for the TP, with PCCs greater than 0.70 for both seasons during day- and nighttime (Figs. S2 and S3), largely attributed to the high spatial quality of CMFD, which we used to drive the model. Interestingly, CLM5.0 accurately simulated land surface emissivity with a small RMSE (0.01) and average bias (<0.01) when compared to MODIS data (Fig. S4). Overall, CLM5.0 could reasonably reproduced TP LST in winter and summer, but with large biases in the magnitudes, indicating large rooms for improvement in TP LST simulation.

### Impact of sensible heat roughness length on daytime LST

**Figure 2:**
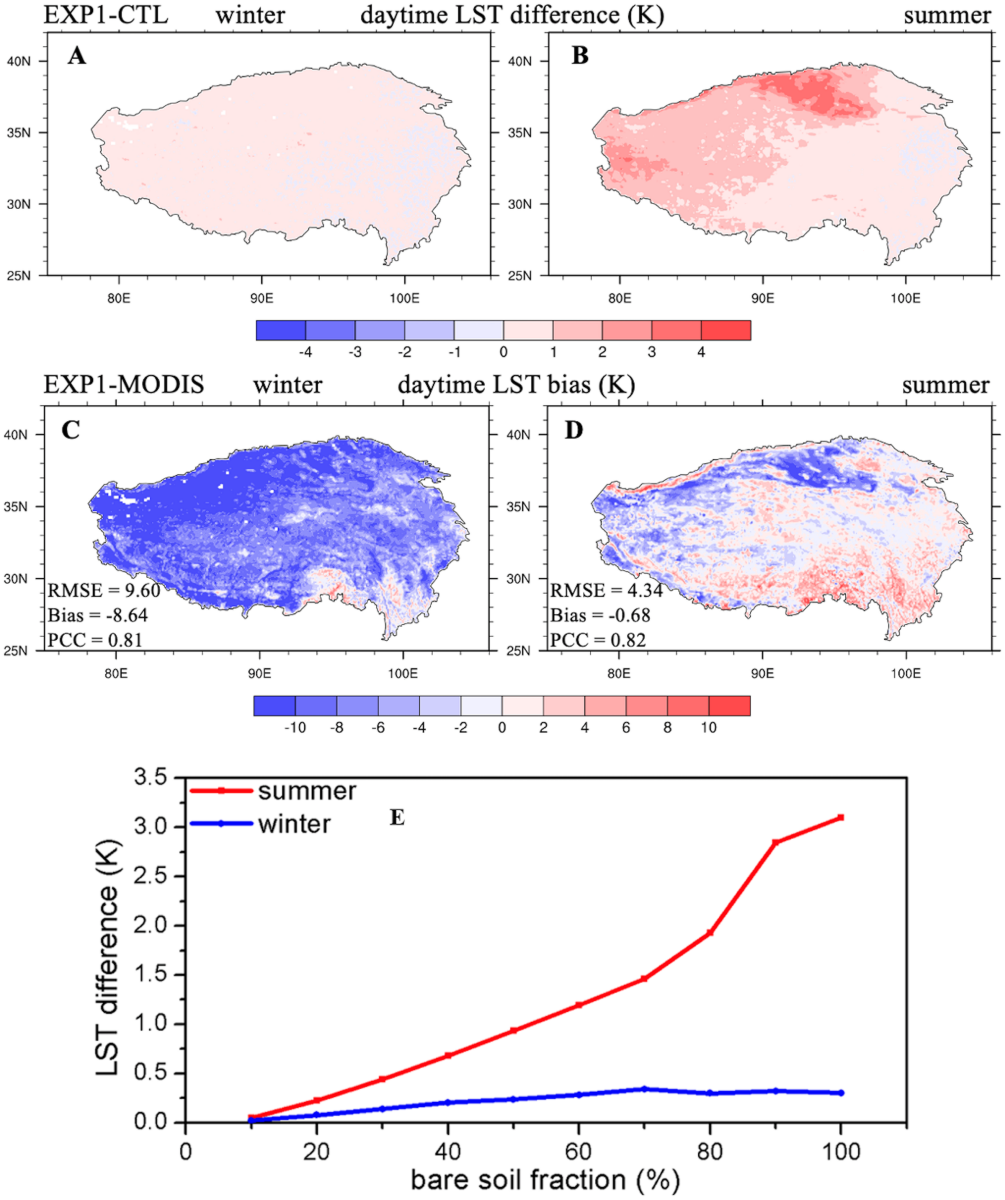
Seasonal distributions of (A, B) the daytime LST difference (unit: K) between EXP1 and CTL, (C, D) EXP1 LST bias compared with MODIS/Aqua data, and (E) LST difference variations with bare soil fraction averaged over 2003–2018 for winter and summer.

We applied the revision of *z*_0*h*,*g*_ (‘Revision of ground sensible heat roughness length scheme’) in CLM5.0, and the impact of this revision on LST for the TP is shown in Fig. 2. The EXP1 simulation increased the daytime LST compared with that of CTL simulations over some regions with bare-soil-underlying conditions, such as the Qaidam Basin and western TP (Figs. 2A, 2B), and the LST increases were more significant when the bare soil fraction increased, especially in summer (Fig. 2E). This mainly occurred due to the revision scheme (Eq. (6)), which reduced *z*_0*h*,*g*_values over the same bare-ground surface, and then decreased the sensible heat flux; therefore, it increased daytime LST, which is consistent with the results of Wang et al. (2014). The EXP1 simulation reduced cold daytime biases in the CTL simulation over bare ground surface: the RMSE was reduced from 9.77 to 9.60 K in winter (Fig. 2C), and from 5.03 to 4.34 K in summer (Fig. 2D), demonstrating the important role of *z*_0*h*,*g*_ over bare-ground surface. Improvements in nighttime LST in this study with the revision of *z*_0*h*,*g*_were negligible (Fig. S5) due to the little impact of the *z*_0*h*,*g*_ revision on nighttime sensible heat flux (Zeng, Wang & Wang, 2012).

### Impact of soil thermal conductivity on LST

**Figure 3:**
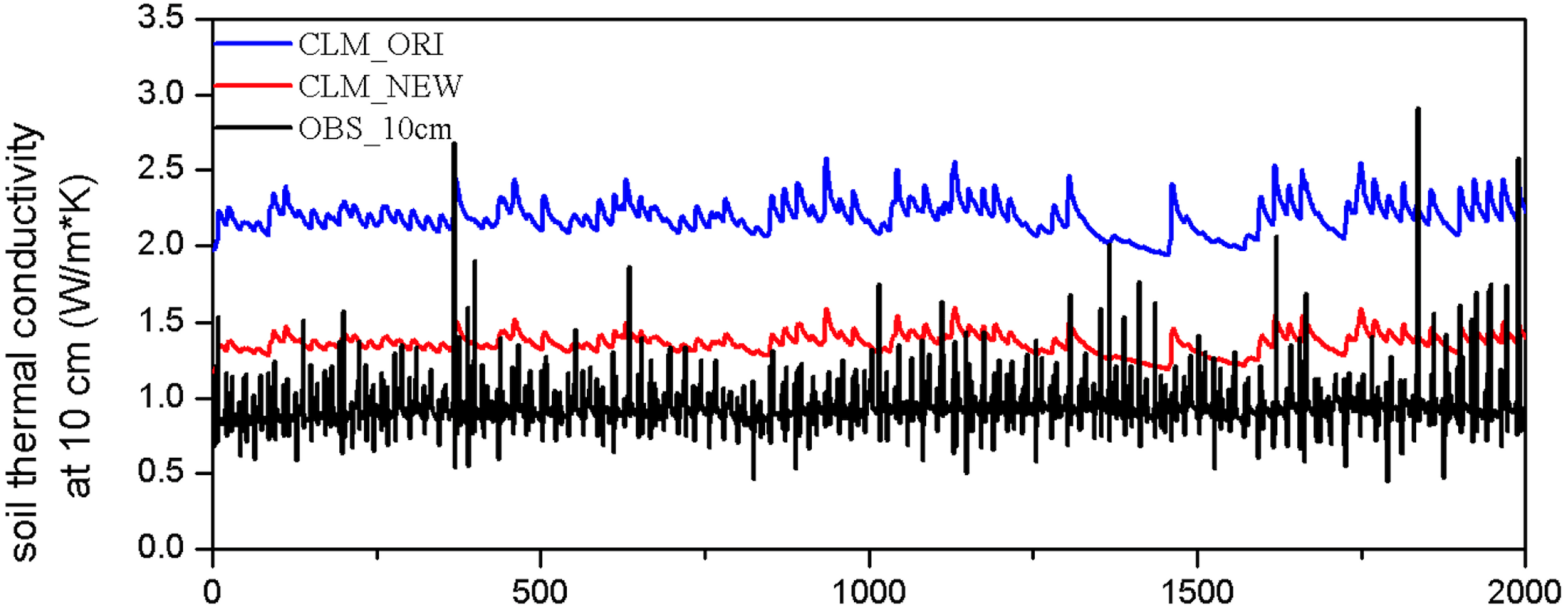
Hourly 10 cm soil thermal conductivity (unit: W m^−1^ K^−1^) from simulated [CLM_*O*_*RI* (blue line) and CLM_NEW (red line)] and in situ observations (black line) at BJ station during summer 2008.

Two soil thermal conductivity parameterizations (‘Model Description’ and ‘Modification scheme for soil thermal conductivity’) were applied to CLM5.0 and evaluated against the BJ station observations, which are referred to hereafter as CLM_ORI and CLM_NEW, respectively. These two offline simulations were driven by observational half-hourly in situ atmospheric forcing data during the summer in 2008. We compared the 10 cm soil thermal conductivity from in situ observations with CLM_ORI and CLM_NEW simulations, as shown in Fig. 3. CLM_ORI largely overestimated the soil thermal conductivity at the BJ site while CLM_NEW effectively reduced the average bias with CLM_ORI from 1.11 to 0.26 W m^−1^ K^−1^. The slight overestimations in CLM_NEW may be related to the inaccurate prediction of soil moisture (Pan et al., 2017).

**Figure 4:**
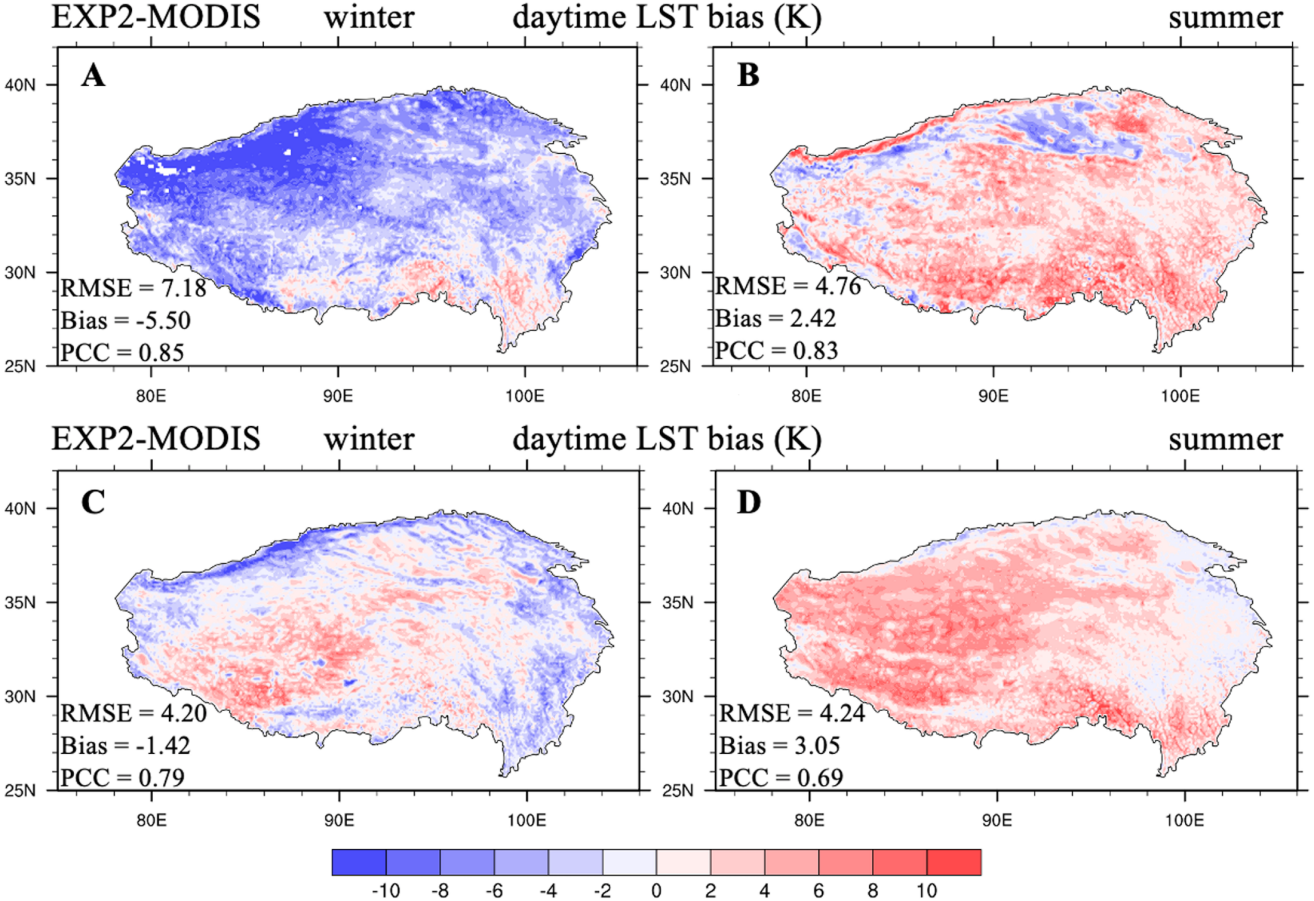
Seasonal distributions of the (A, B) daytime and (C, D) nighttime LST bias (unit: K) between EXP2 and MODIS/Aqua (EXP2-MODIS) averaged over 2003-2018 for winter and summer.

Next, this modified soil thermal conductivity parameterization (‘Modification scheme for soil thermal conductivity’) was implemented in CLM5.0 to explore the impact of soil thermal conductivity on LST for the whole TP during day- and nighttime. Figure 4 shows the spatial distribution of day- and nighttime LST biases between EXP2 and MODIS Aqua data averaged from 2003 through 2018 in winter and summer. The biases presented large spatial and diurnal variations. In winter, cold biases (average bias: −5.50 K) were dominant over most of the TP during daytime, whereas warm biases appeared over the western TP, and cold biases were distributed along the TP edges during nighttime (Fig. 4C). In summer, warm biases were occurred for the whole TP during both daytime and nighttime, with the average bias values of 2.42 and 3.05 K, respectively (Figs. 4B, 4D). Compared to CTL, EXP2 considerably much improved the TP LST simulation during both the daytime and nighttime for the two seasons (LST RMSE: daytime: 9.77 compared to 7.18 K in winter, 5.03 compared to 4.76 K in summer; nighttime: 4.85 compared to 4.20 K in winter, 6.99 compared to 4.24 K in summer; Table 3). After modification, lower soil thermal conductivity permitted less energy transfer from the land surface to deep soil during daytime, resulting in more sustained energy on the surface, producing warming. During the night, less energy transferred from the deep soil to the land surface, leading to a reduction in LST. As a result, the introduction of a more realistic representation of soil thermal conductivity into CLM5.0 significantly improved LST simulations during day- and nighttime in the two seasons.

**Table 3:** Values of LST average bias and RMSE (unit: K) between four offline CLM5.0 simulations and MODIS/Aqua observations averaged over TP during 2003–2018 for summer and winter during daytime and nighttime.

Statistical metrics		Winter		Summer	
		Average bias	RMSE	Average bias	RMSE
Daytime (K)	CTL	−8.81	9.77	−1.61	5.03
	EXP1	−8.64	9.60	−0.68	4.34
	EXP2	−5.50	7.18	2.42	4.76
	EXP3	−4.61	6.47	1.39	4.30
Nighttime (K)	CTL	2.27	4.85	6.43	6.99
	EXP1	2.27	4.85	6.43	6.99
	EXP2	−1.42	4.20	3.05	4.24
	EXP3	−1.40	3.80	2.25	3.40

### Impact of soil evaporation resistance on LST

**Figure 5:**
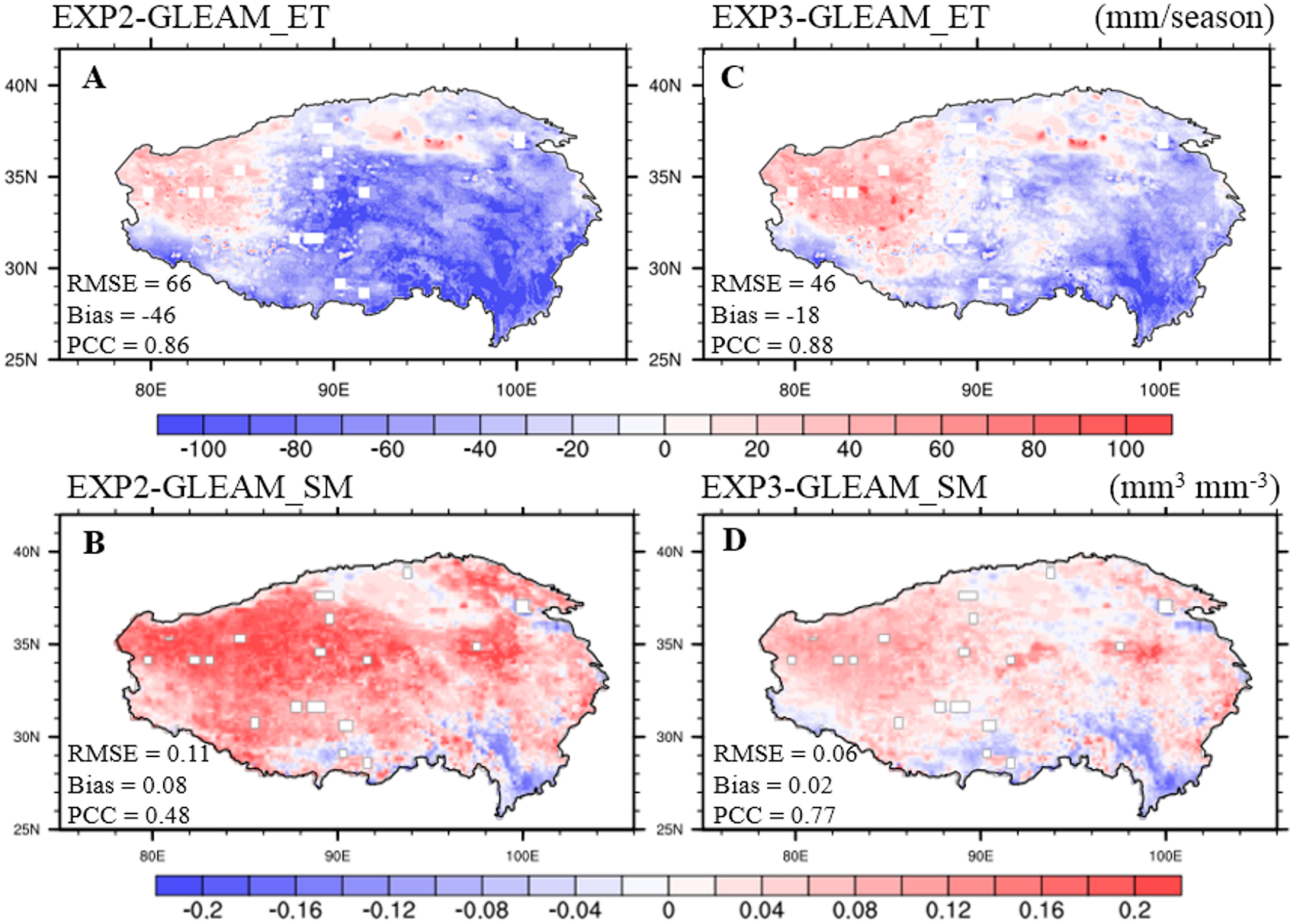
Seasonal distributions of the (A, C) ET (unit: mm/season) and (B, D) surface soil moisture (unit: mm^3^ mm^−3^) biases between EXP2, EXP3, and GLEAM averaged over 2003–2018 for summer.

Next, we further evaluated and improved the performance of CLM5.0 on simulated ET and surface SM during summer. Figure 5A plots the spatial distribution of seasonal ET differences between EXP2 and GLEAM_ET data averaged over the summers of 2003-2018. EXP2 underestimated summer ET for almost the entire TP, except for the Qaidam Basin and western TP, with an average bias of −46 mm/season (although simulated summer ET showed a similar spatial distribution patterns to those from GLEAM_ET data; PCC = 0.86). Such underestimation of ET leads to wetter soil in 87.6% of TP regions, with an average bias value of 0.08 mm^3^ mm^−3^ for SM when compared with GLEAM_SM data (Fig. 5B) because soil evaporation acted as the mainly component of ET (LAI values were generally well below 1.0 in the central and western parts of TP; Fig. S6). In addition, we found that summer ET was underestimated and SM was overestimated with EXP2 compared to other remote sensing observations (Figs. S7 and S8). The underestimation of ET (soil evaporation) and the prediction of wetter surface soil were improved by the modification of the soil evaporation resistance scheme as described in ‘Modification of soil evaporation resistance scheme’. We compared the ET simulated with EXP3 to that with EXP2 and to observed GLEAM_ET data. EXP3 generated more seasonal summer ET across the TP (red areas in Fig. S9B) than EXP2, especially in central TP. Moreover, seasonal summer ET estimated in EXP3 agreed better with GLEAM_ET data (Fig. 5C), with a smaller ET average bias (−18 mm/season) and larger ET PCC (0.88). The larger seasonal summer ET (soil evaporation) from EXP3 led to less soil water content when compared with the SM from EXP2 (blue parts in Fig. S9C), which was closer to the GLEAM_SM data (Fig. 5D), as EXP3 produced smaller SM average bias (0.02 mm^3^ mm^−3^) and larger SM PCC (0.29 larger than EXP2).

**Figure 6:**
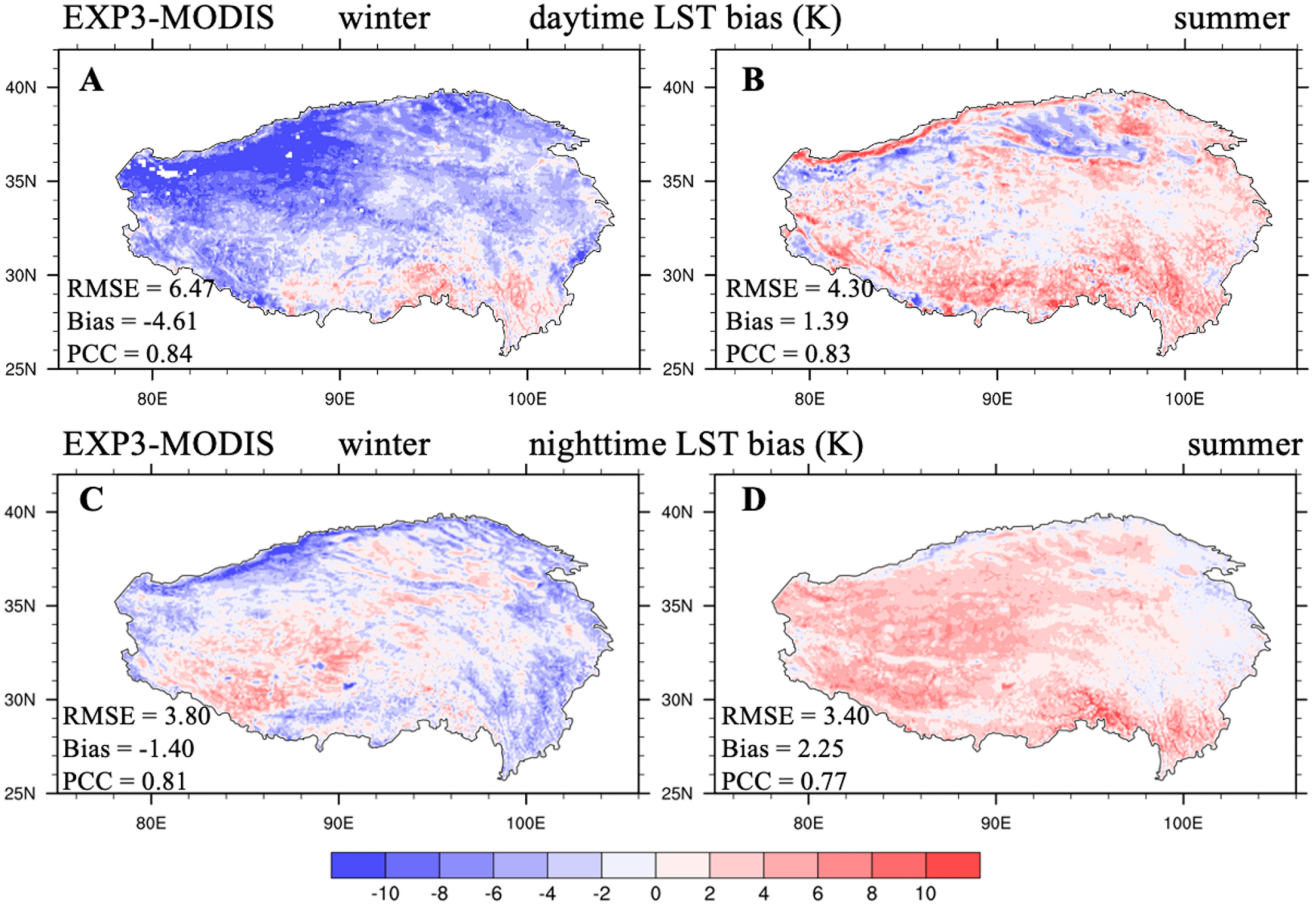
Seasonal distributions of the (A, B) daytime and (C, D) nighttime LST bias (unit: K) between EXP3 and MODIS/Aqua (EXP3-MODIS) averaged over 2003–2018 for winter and summer.

The larger seasonal summer ET from EXP3 implied that the lower daytime LST was due to the evaporation cooling effect when compared with daytime LST from EXP2 (Fig. S10B), being closer to MODIS daytime LST during summer, with an RMSE of 4.30 K (Fig. 6B). The lower SM from EXP3 corresponded to a smaller soil thermal inertia, implying less heat transfer from deep soil to the land surface during nighttime, producing lower nighttime LST when compared with that from EXP2 (Fig. S10D), being closer to MODIS nighttime LST during summer, with an RMSE of 3.40 K (Fig. 6D). In addition, the modification of the soil evaporation resistance scheme had a negligible impact on winter ET (Fig. S9A), but the lower soil liquid water content simulated by EXP3 in summer led to less soil ice content, and then to lower soil thermal conductivity in winter. Thus, EXP3 predicted higher daytime LST and lower nighttime LST during winter when compared to that of EXP2 (Figs. S10A, S10C). Furthermore, EXP3 produced better daytime and nighttime LST in winter when compared with MODIS observations (Figs. 6A, 6C; LST RMSE: 6.47 compared to 7.18 K in daytime, 3.80 compared to 4.20 K in nighttime).

Table 3 summarizes the LST average bias and RMSE between the four offline CLM5.0 simulations and MODIS/Aqua observations averaged over the TP during daytime and nighttime. Adjustments to these three schemes (ground sensible heat roughness length, soil thermal conductivity, and soil evaporation resistance) improved the simulations of the diurnal LST cycle for the TP during winter and summer.

## Discussion

In this study, the performance of CLM5.0 when simulating diurnal LST variations was evaluated and further improved for the whole TP by introducing the ground sensible heat roughness length formulation from Zeng, Wang & Wang (2012), the recommended soil thermal conductivity scheme from Dai et al. (2019), which considers the volumetric fractions of SOM and soil gravel, and a modified soil evaporation resistance parameterization that is appropriate for the soil texture of the TP. Nevertheless, significant systematic deviations in the LST were still existed between simulations from CLM5.0 and observations from MODIS remote sensing (Fig. 6). The above analysis and previous studies help to initially confirm some possible reasons for these deviations.

**Figure 7:**
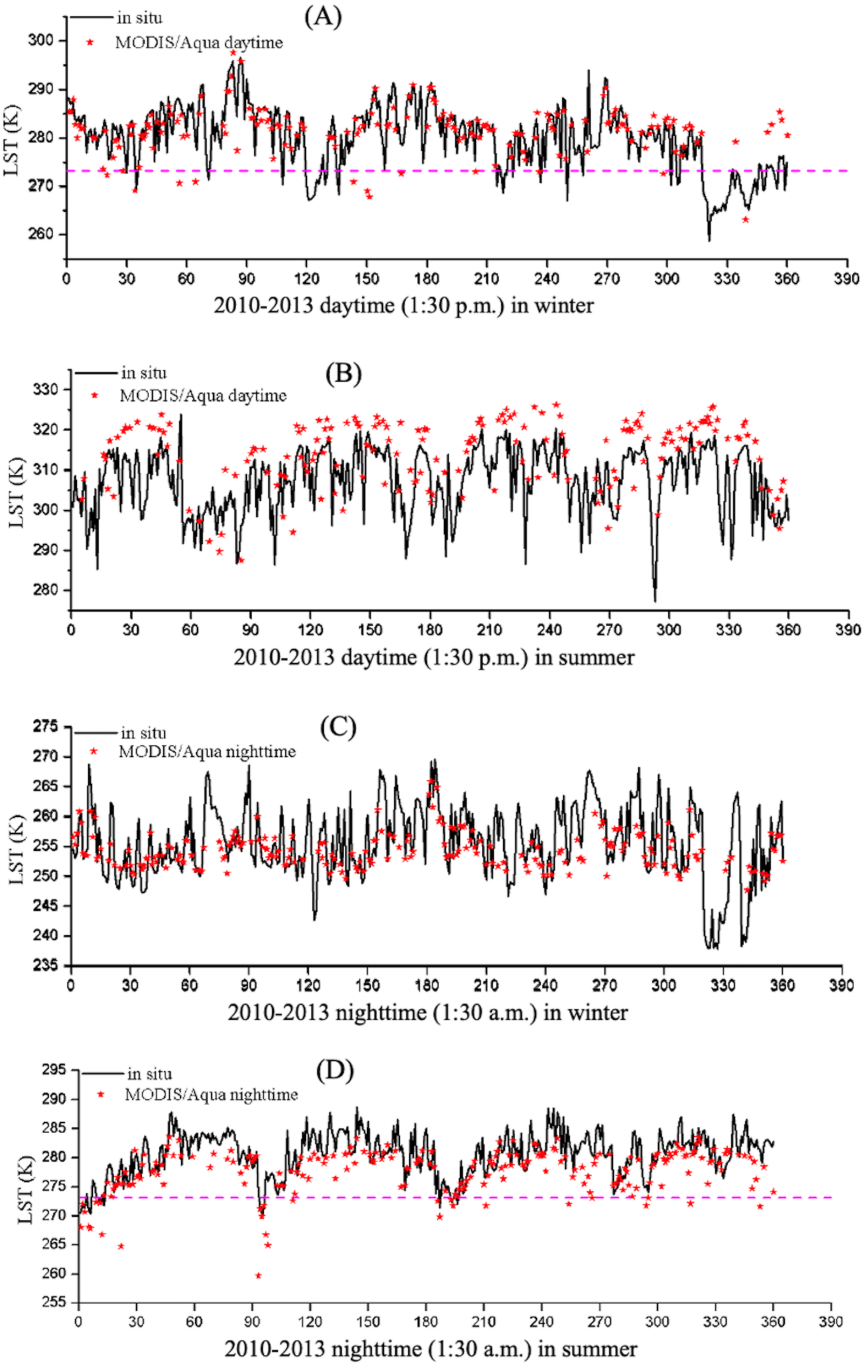
Comparison of the NASDE station LST (unit: K) between the MODIS/Aqua and *in situ* observations for (A, B) 1:30 p.m. and (C, D) 1:30 a.m. per day of winter and summer during 2010–2013.

First, the uncertainties of the satellite remote sensing LST data. A previous study evaluated the accuracy of the MODIS LST product and found that the bias was less than 1 K, with the exception of the bare-ground land cover type (Duan et al., 2019). Figure 7 displays the results of MODIS LST derived from Aqua observations versus the ground-based LST for daytime (1:30 p.m.) and nighttime (1:30 a.m.) at the NASDE station for summer and winter during 2010–2013. Only the highest-quality MODIS LST data (LST mean errors less than 1 K) marked in Wan (2013) were selected for evaluation. We found that the MODIS/Aqua products can well-capture the values and temporal variations in daytime and nighttime LSTs at NASDE station for both seasons, although the MODIS/Aqua products overestimated (underestimated) the LST during summer daytime (nighttime). The errors of MODIS LST products may be caused by: (1) the spatial inconsistency, which is 0.05^o^ (∼5, 600 m) for MODIS products and single-point for ground-based LST; (2) the uncertainty in the determination of surface emissivity, which strongly influences the derived of LST (Wan et al., 2002; Wang et al., 2014). The accuracy of MODIS surface emissivity retrieval depends on the accuracy of the land cover type product, and the error in surface emissivity was caused by the lack of global representativeness of land cover type (Duan et al., 2019).

**Figure 8:**
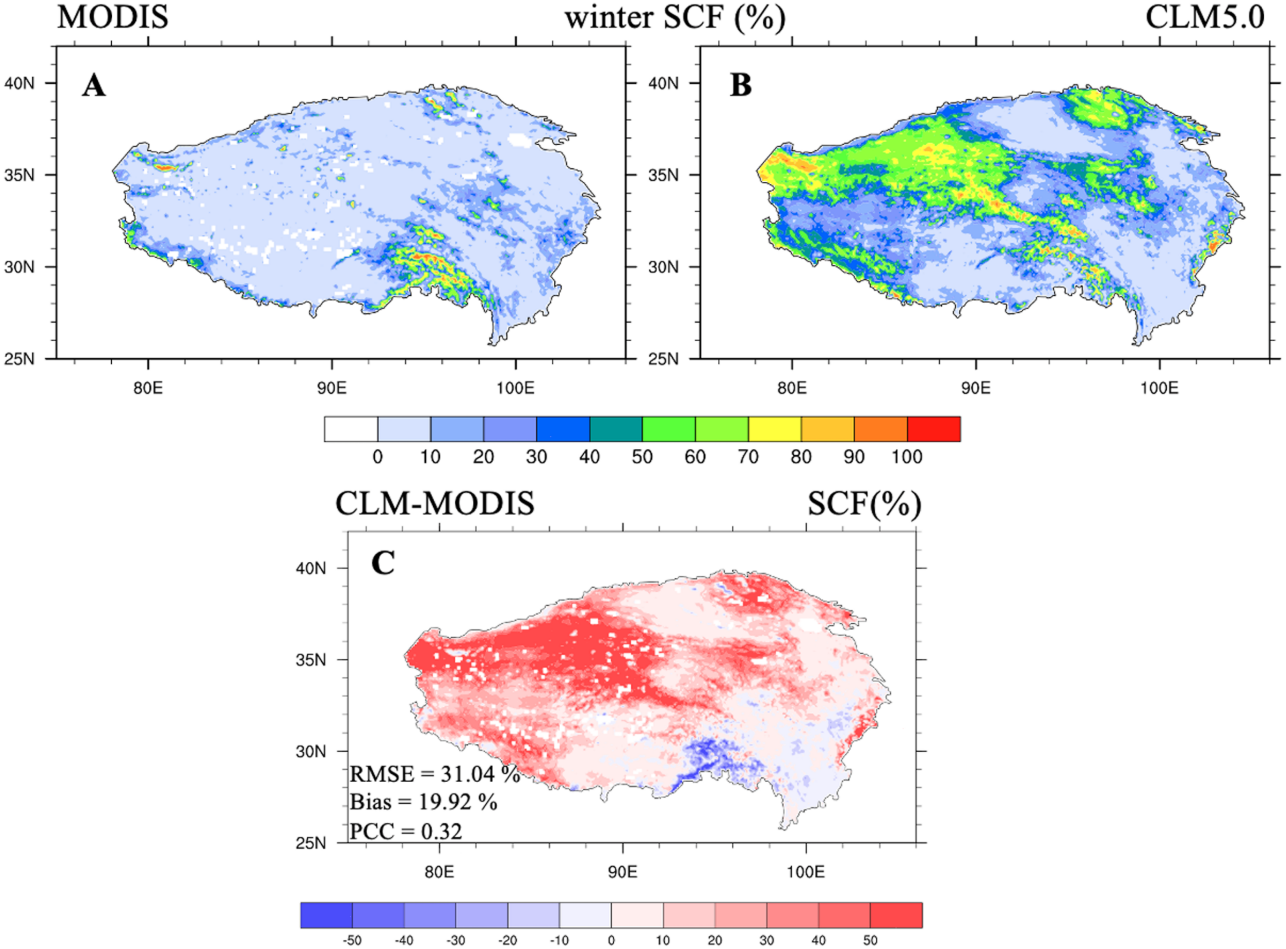
Snow cover fraction (unit: %) distributions from (A) MODIS data, (B) CLM5.0 simulations, and (C) snow cover fraction errors between CLM5.0 and MODIS data averaged over 2003–2018 for winter.

Second, the deficiencies in the simulated CLM5.0 snow cover fraction (SCF) lead to biases in simulated LST, especially for the cold biases during winter daytime. Figure 8 shows the spatial distribution of the SCF from the MODIS satellite remote sensing observations, the CLM5.0 simulated SCF, and SCF errors from CLM5.0 during winter for 2003 through 2018. The SCF over the northwestern TP was clearly overestimated, especially in the Kunlun and Qilian Mountains (RMSE = 31.04%, average bias = 19.92%). A higher SCF leads to higher surface albedo, absorbing less solar radiation, and resulting in lower daytime LST during winter (Fig. 6A). The large SCF biases from CLM5.0 simulation may have come from: (1) the model splitting total input precipitation between snowfall and rainfall according to empirical formulation only based on air temperature (Lawrence et al., 2018), which may have introduced biases in the simulation of the SCF (Dai, 2008; Ding et al., 2014; Guo et al., 2018); (2) the SCF parameterization in CLM5.0. A new scheme (Swenson & Lawrence, 2012) has been used since CLM4.5 to capture the seasonal snow depth-SCF evolution. However, the parameterization scheme cannot capture the effects of the very complex terrain structures of the northwestern and southeastern TP on the SCF, and the fixed snow accumulation factor may have also led to inaccurate SCF simulations (Swenson & Lawrence, 2012; Wang et al., 2020).

**Figure 9:**
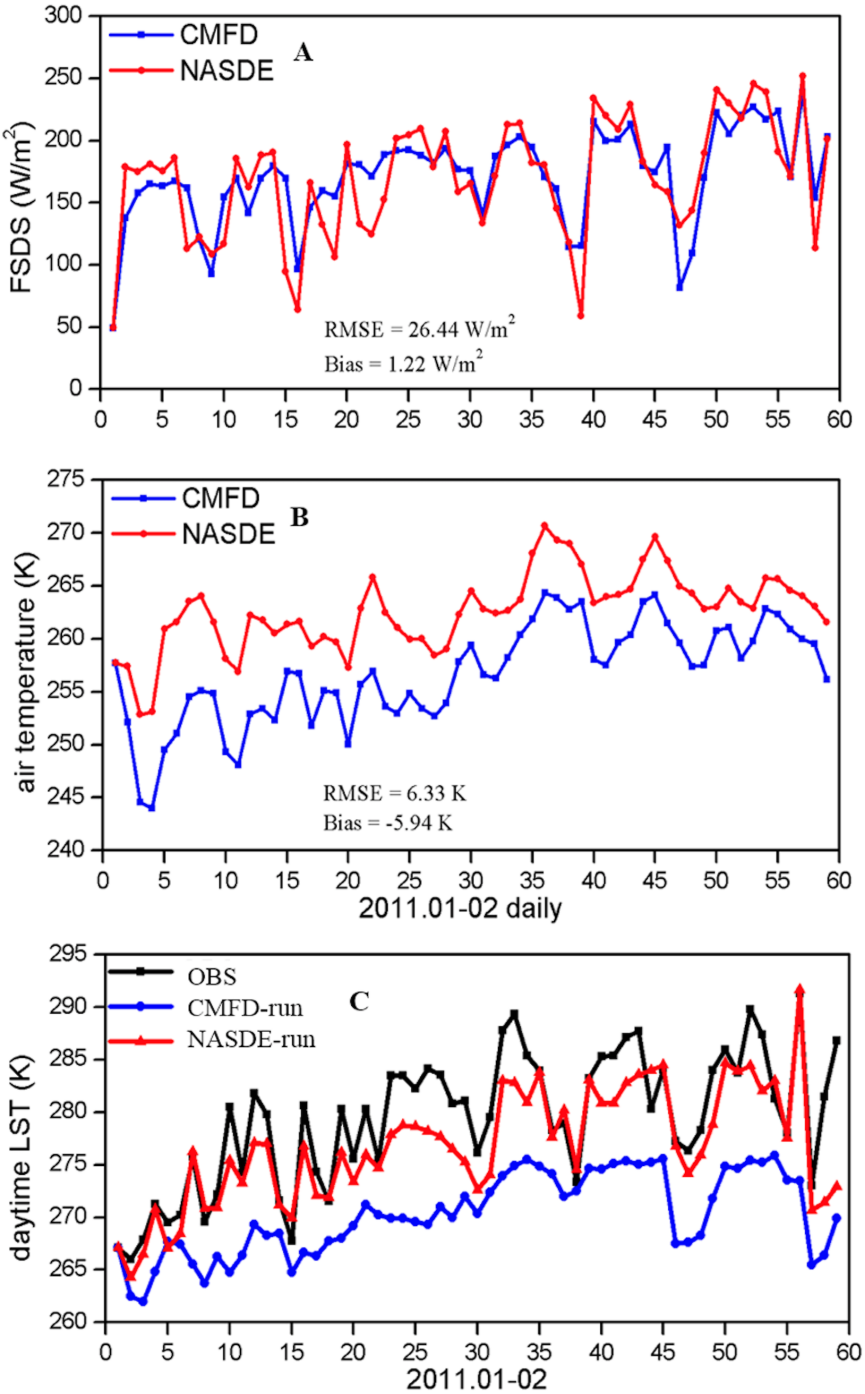
Time series of (A) daily downward shortwave radiation, (B) daily air temperature from CMFD forcing (blue line) and NASDE observations (red line); (C) daytime LST for CMFD-run (blue line), NASDE-run (red line), and NASDE observations (black line) during January through February 2011.

Third, there were deficiencies in the atmospheric forcing dataset that led to biases in the simulated LST. The CMFD benefits from merging from observations from 753 CMA operational stations, and it was the best-available atmospheric forcing dataset in China at the time (He et al., 2020). However, the distribution of these operational CMA stations shows large spatial variations, with almost no stations are in the northwestern TP, which increases the likelihood of large errors in the CMFD over this region (Li et al., 2018). Figures 9A, 9B shows that the daily downward shortwave radiation from CMFD was almost equal to NASDE observations, but the 2-m air temperature was lower than that from NASDE. We conducted two simulations: CMFD-run and NASDE-run, which were forced with CMFD and NASDE observational air temperature (other variables were still obtained from CMFD), respectively. A time series of the daytime LST values is depicted in Fig. 9C for the two simulations and observations from January through February 2011. Large daytime cold biases occurred when the model was forced by CMFD, and LST was improved by forcing with more accurate observational data (LST RMSE: 10.02 K for CMFD-run and 4.00 K for NASDE-run). These atmospheric forcing data biases affected the simulated surface energy variables. However, they are difficult to acquire for the entire TP areas for in situ observations, even though they may improve the simulations.

## Conclusions

LST is an important variable in the surface energy budget and the energy exchanges between the land surface and atmosphere. However, the lack of a whole region’s long-term ground-based observations for whole regions is a barrier to understanding the important role of LST in the land surface processes of the entire TP. Thus, remote sensing observations and LSMs are employed to produce long-term LST records in a continuous spatiotemporal scale.

In this work, we employed in situ observations, MODIS remoter sensing LST and surface emissivity products, and GLEAM ET and SM products to evaluate the ability of CLM5.0 to simulate the diurnal LST cycle for the whole TP. The results showed that the LST biases display large spatial and diurnal variations: (1) during the daytime, cold biases were dominant over most of the TP in winter, whereas areas with negative LST biases were mainly observed for bare-ground regions in summer; (2) during the nighttime, areas with positive LST biases covered the whole TP for both seasons. These large biases in LST encouraged us to improve the ability of CLM5.0 to simulate diurnal LST variations by modifying the computation of the surface energy balance.

Three modifications to the model physics were investigated to resolve the above LST deficiencies: (1) the revision of ground sensible heat roughness length (*z*_0*h*,*g*_) from Zeng, Wang & Wang (2012) was implemented into CLM5.0; (2) the recommended soil thermal conductivity scheme from Dai et al. (2019), which was formulated with volumetric SOM and soil gravel fractions; and (3) a modification of soil evaporation resistance parameterization that was more suitable for the sandier soil of the TP. Four numerical experiments were designed to assess the impact of these three above modifications on model performance.

The revision of *z*_0*h*,*g*_ in CLM5.0 reduced the cold biases in the daytime over bare soil regions (Qaidam Basin and western TP), but its effects were negligible at nighttime. The recommended soil thermal conductivity scheme obviously improved the calculation of soil thermal conductivity compared with in situ observations (‘Impact of soil thermal conductivity on LST’). The regional application of modified soil thermal conductivity parameterization in CLM5.0 significantly improved the simulated LST during daytime and nighttime. The modification of soil evaporation resistance parameterization increased the soil evaporation, reproduced more accurate ET and SM compared with GLEAM products, and further improved diurnal LST variations in both seasons. In summary, the improvements in the simulated LST resulting from EXP2 and EXP3 highlight the importance of the effects of soil texture on soil thermal properties and soil evaporation, and further indicat the crucial role of accurate soil texture information in determining the land surface energy and water budget.

Three factors were discussed to investigate the possible reasons associated with the LST biases between the model simulations and MODIS observations: the uncertainties in the satellite remote sensing LST data, the deficiencies in the simulated SCF, and the atmospheric forcing data. It is unclear which factor is dominant. Regardless, accurate soil texture information and more realistic soil thermal conductivity and soil evaporation resistance parameterization schemes significantly influence the accuracy of the simulated LST. This work showed that remotely sensed LST has the potential to be used in CLM5.0 simulations to evaluated diurnal LST variations over the entire TP, which may further help to assess model parameters and land surface schemes.

##  Supplemental Information

10.7717/peerj.11040/supp-1 Supplemental Information 1 Soil texture, land surface temperature simulations, and evapotranspiration over the Tibetan Plateau data

10.7717/peerj.11040/supp-2 Supplemental Information 2 Raw data of the soil thermal conductivity from the simulated (CLM_ORI and CLM_NEW) and in situ observations at BJ station during summer 2008, and preparation for Fig. 3

10.7717/peerj.11040/supp-3 Supplemental Information 3 Raw data of the daily downward shortwave radiation and daily air temperature from CMFD forcing and NASDE observations. In addition, raw data of the daytime LST for CMFD-run, NASDE-run, and NASDE observations, and preparation for Fig. 9

## Additional Information and Declarations

### Data Availability

The following information was supplied regarding data availability: Raw measurements are available in Supplementary Files.
