# Supplementary material for: Evaluating and improving simulations of diurnal variation in land surface temperature with the Community Land Model for the Tibetan Plateau
Source: PeerJ. 2021 Mar 16;9:e11040. doi: 10.7717/peerj.11040 (PMC7977383; doi:10.7717/peerj.11040)
Supplement: Supplemental Information 1 [file peerj-09-11040-s001.docx]

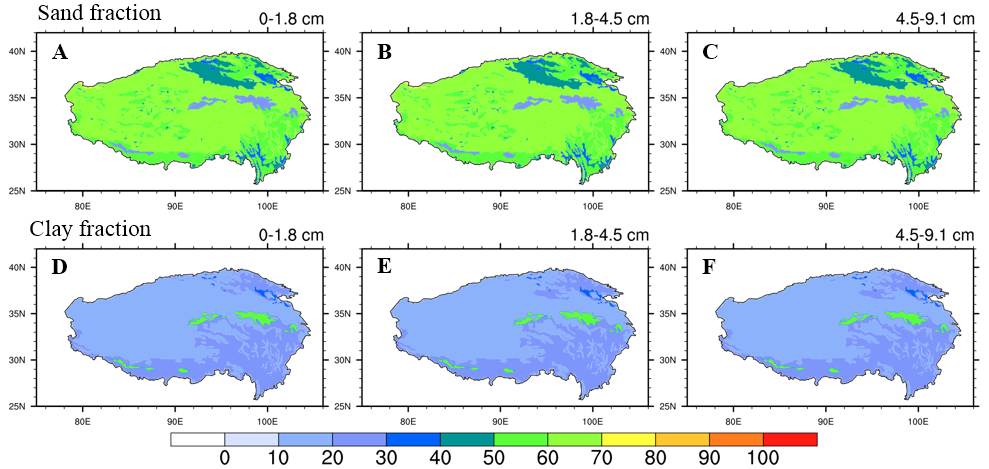


**Figure S1** Surface (0-10 cm) soil (A-C) sand and (D-F) clay fraction (unit: %) over the TP (from CLM5.0 surface data).


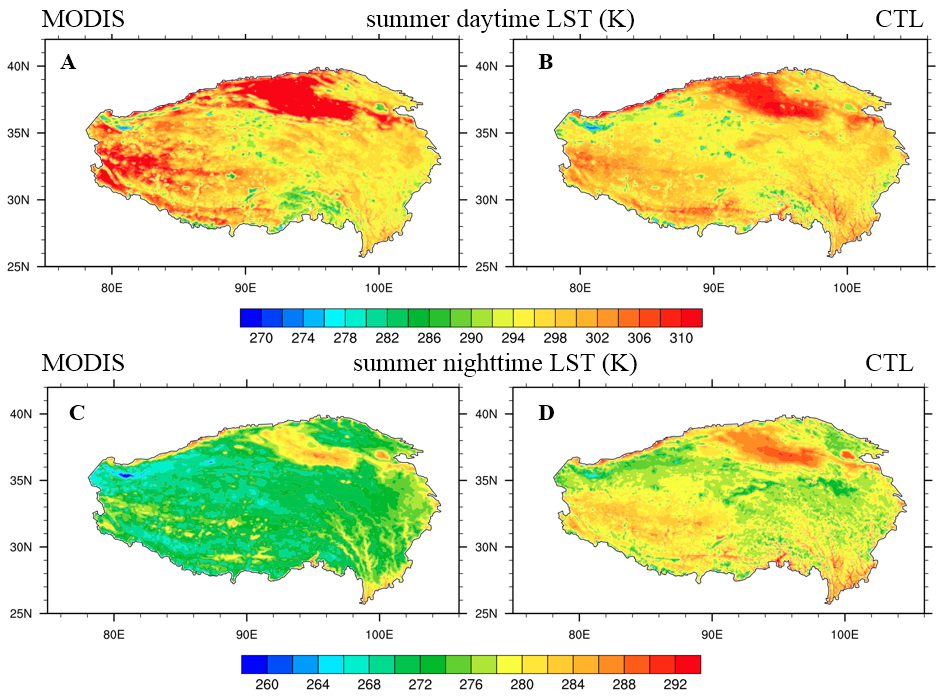


**Figure S2** Spatial distribution of (A, B) daytime and (C, D) nighttime land surface temperature (unit: K) averaged over summers of 2003-2018 from MODIS/Aqua and CTL simulations.


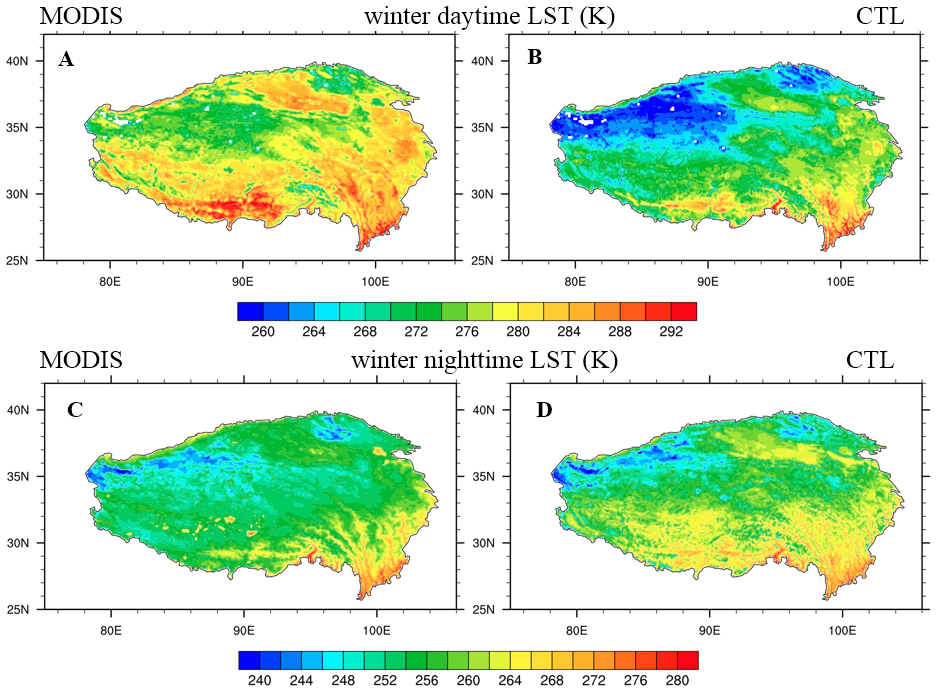


**Figure S3** Spatial distribution of (A, B) daytime and (C, D) nighttime land surface temperature (unit: K) averaged over winters of 2003-2018 from MODIS/Aqua and CTL simulations.


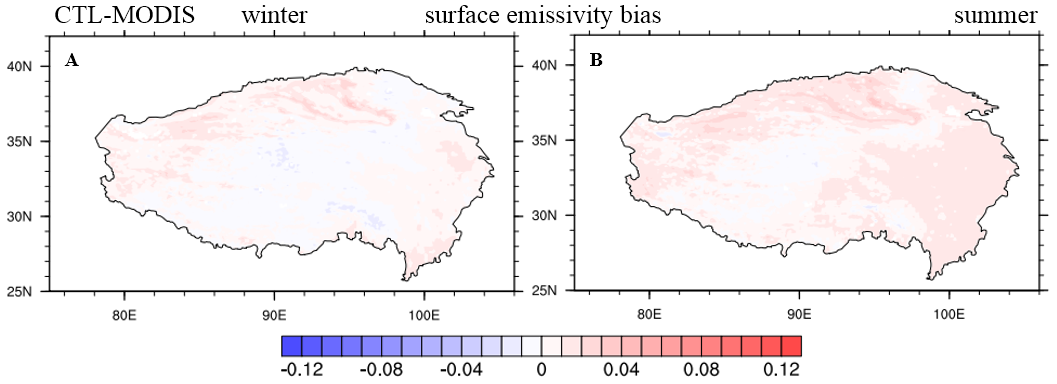


**Figure S4** Seasonal distributions of the surface emissivity bias between CTL and MODIS/Aqua (CTL-MODIS) averaged over 2003-2018 for (A) winter and (B) summer.


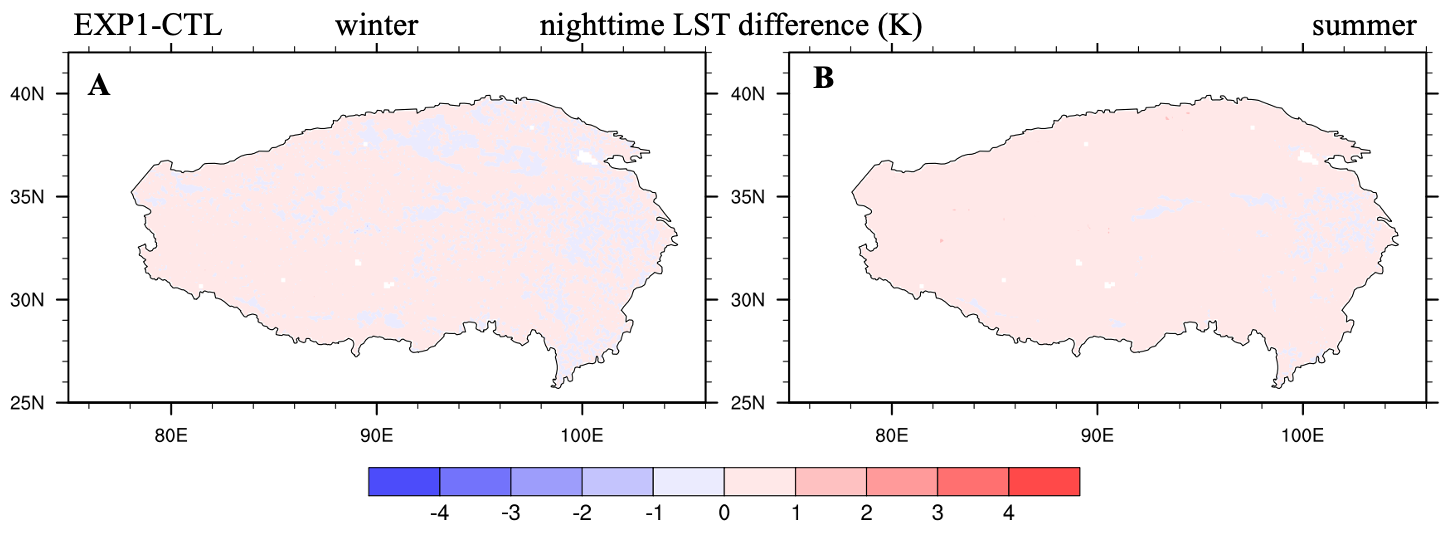


**Figure S5** Seasonal distributions of the nighttime LST difference (unit: K) between EXP1 and CTL averaged over 2003-2018 for (A) winter and (B) summer.


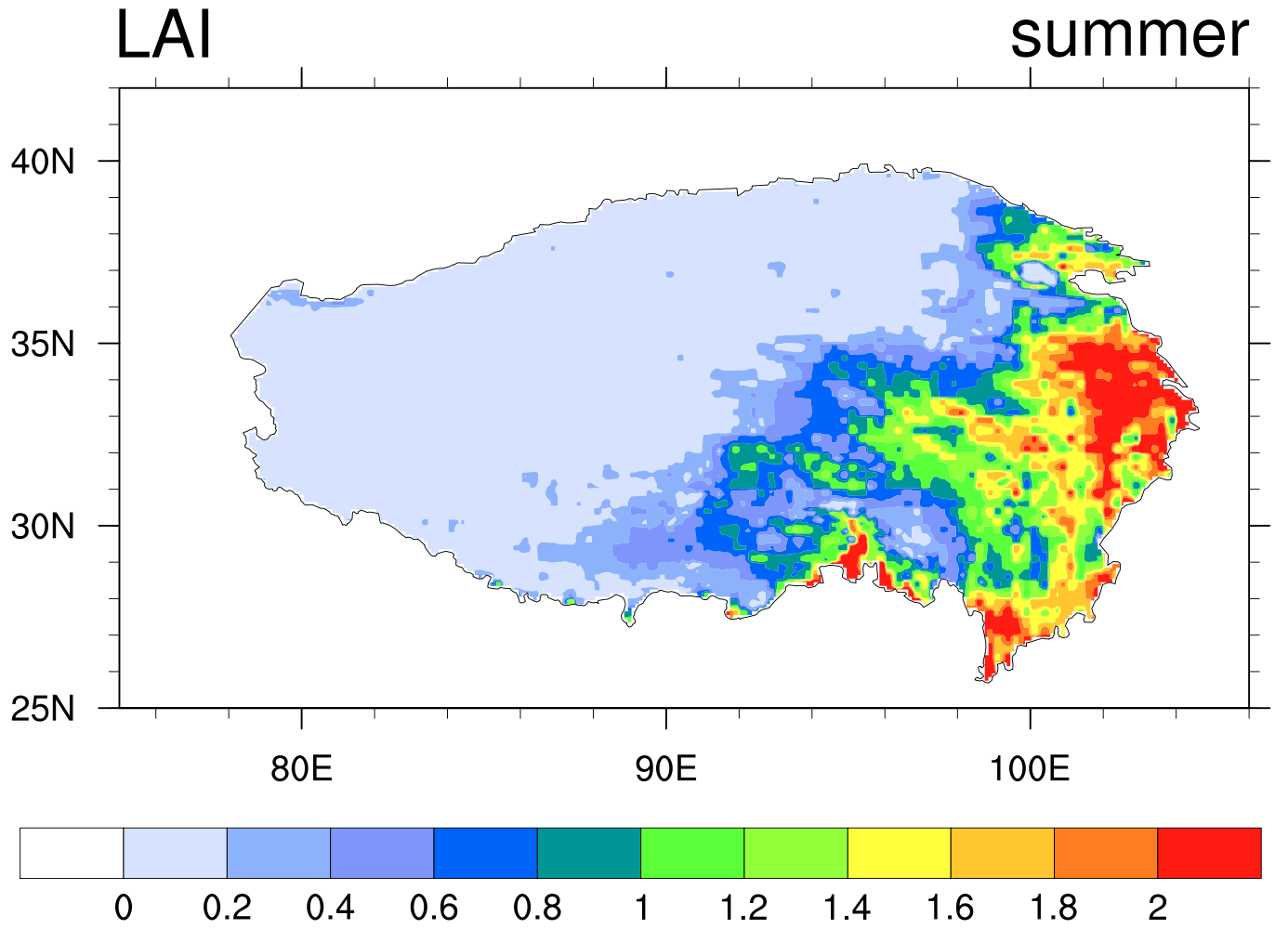


**Figure S6** Spatial distribution of summer leaf area index (LAI) over the TP (from CLM5.0 surface data).


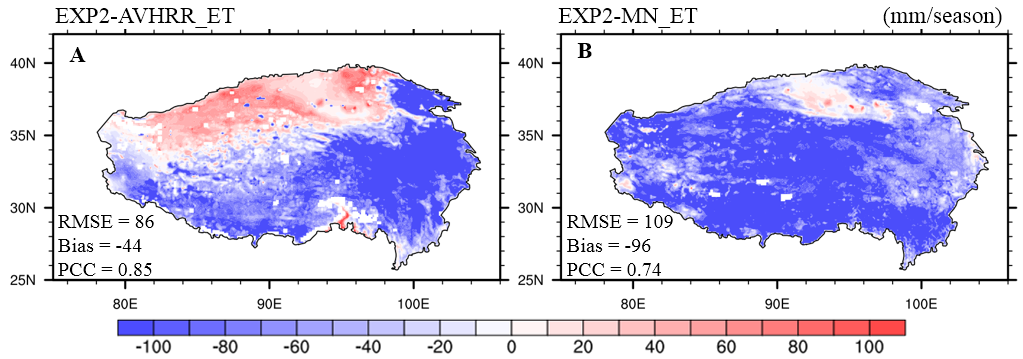


**Figure S7** Seasonal distributions of the ET biases (unit: mm/season) between EXP2, (A) AVHRR_ET ^1^ and (B) MN_ET ^2^ products averaged over 2003-2018 for summer.

^1^ AVHRR_ET data are from Zhang et al. (2010)

^2^ MN_ET data are from Ma et al. (2019)

Zhang K, Kimball JS, Nemani RR, Running SW. 2010. A continuous satellite–derived global record of land surface evapotranspiration from 1983 to 2006. Water Resources Research 46: W09522 DOI 10.1029/2009WR008800.

Ma N, Szilagyi J, Zhang Y, Liu W. 2019. Complementary–relationship–based modeling of terrestrial evapotranspiration across China during 1982–2012: validations and spatiotemporal analyses. Journal of Geophysical Research: Atmospheres 124:4326–4351.


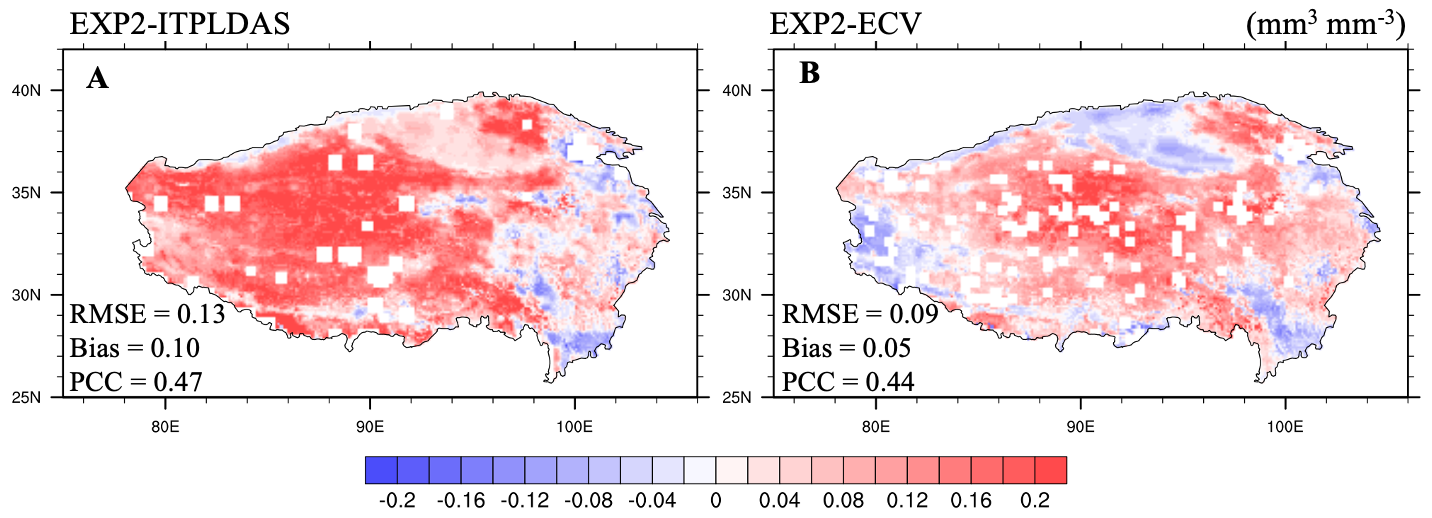


**Figure S8** Seasonal distributions of the 0-5 cm soil moisture biases (unit: mm^3^ mm^-3^) between EXP2, (A) ITPLDAS^3^ and (B) ECV^4^ products averaged over 2003-2011 for summer.

^3^ ITPLDAS data are from Yang et al. (2018)

^4^ ECV data are from Cui et al. (2019)

Yang K. 2018. The soil moisture dataset of China based on microwave data assimilation (2002-2011). National Tibetan Plateau Data Center. DOI 10.11888/AtmosphericPhysics.tpe.249448.file.

Cui Y, Zeng C, Zhou J, Xie H, Wan W, Hu L, Xiong W, Chen X, Fan W, Hong Y. 2019. A spatio-temporal continuous soil moisture dataset over the Tibet Plateau from 2002 to 2015. Scientific Data 6:247 DOI 10.1038/s41597-019-0228-x.


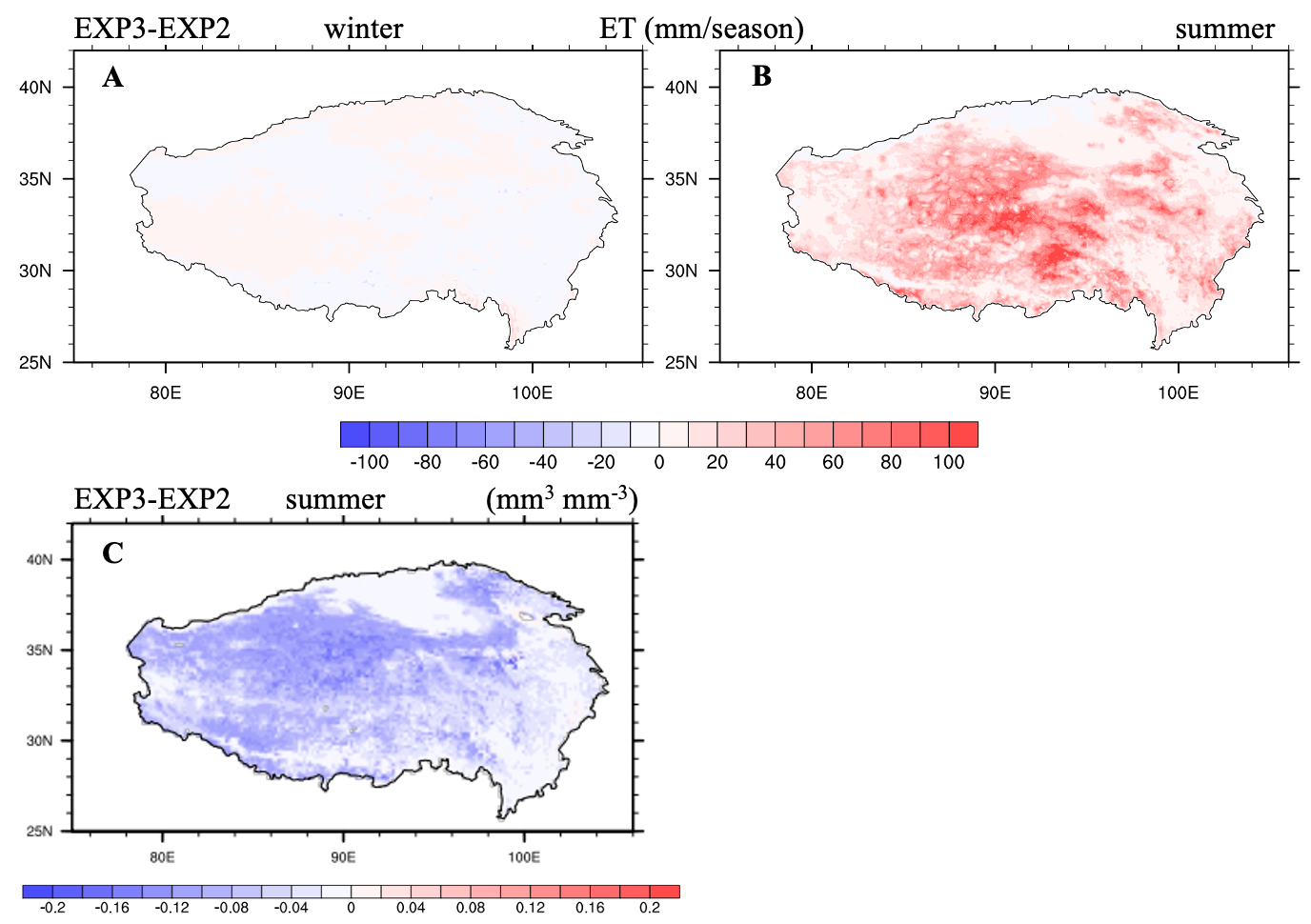


**Figure S9** Seasonal distributions of the (A) winter ET, (B) summer ET (mm/season), and (C) summer surface (0-10 cm) soil moisture (mm^3^ mm^-3^) differences between EXP3 and EXP2 averaged over 2003-2018.


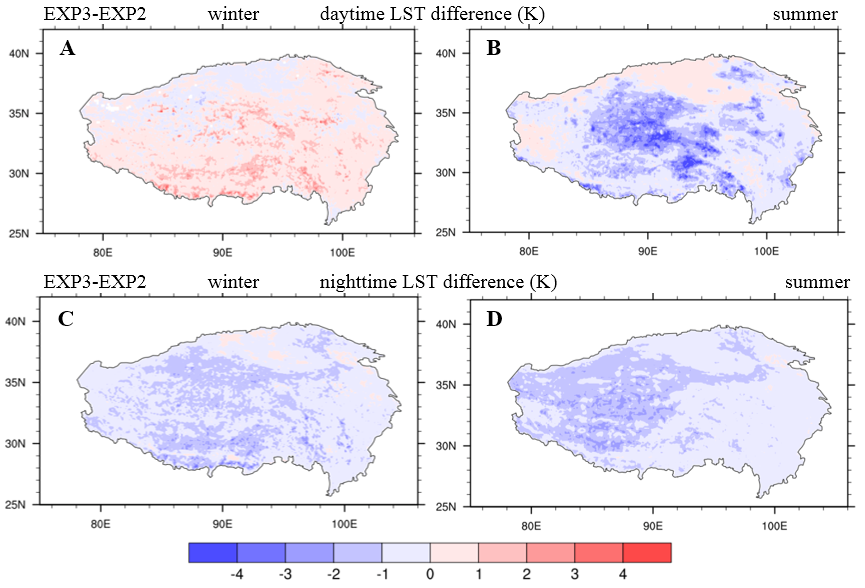


**Figure S10** Seasonal distributions of the (A, B) daytime and (C, D) nighttime LST differences (unit: K) between EXP3 and EXP2 (EXP3-EXP2) averaged over 2003-2018 for winter and summer.


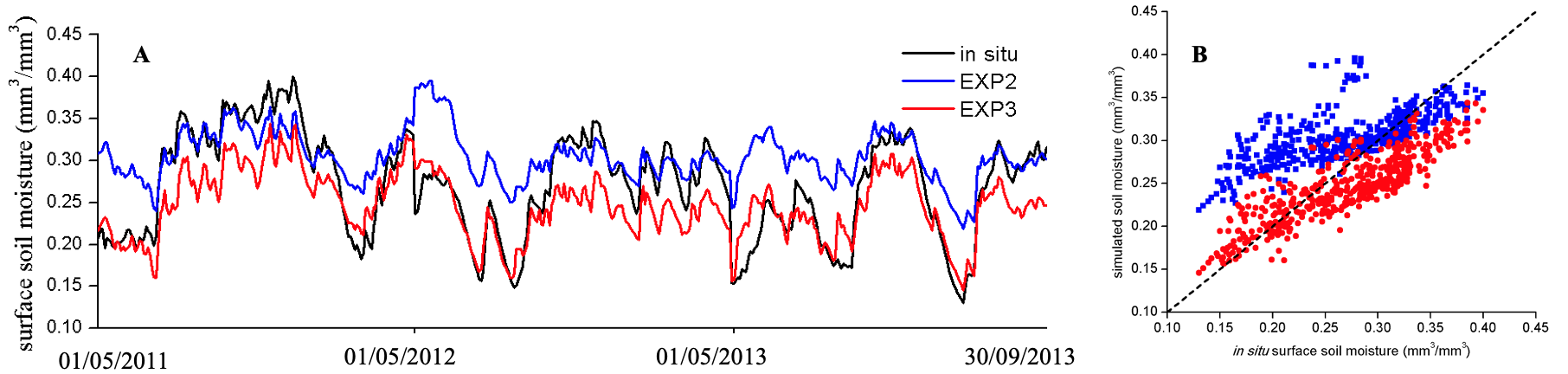


**Figure S11** Comparison of the daily 0-5 cm soil moisture (unit: mm^3^ mm^-3^) from *in situ* observations for the Naqu network (black line) and CLM5.0 simulations [EXP2 (blue line) and EXP3 (red line)] during May-September 2011-2013: (A) the time series and (B) the scatterplot for the Naqu network.
